# The dynamics of ciliogenesis in prepubertal mouse meiosis reveals new clues about testis maturation

**DOI:** 10.3389/fcell.2026.1910205

**Published:** 2026-08-14

**Authors:** I. Pérez-Moreno, P. López-Jiménez, H. Zapata-Polo, S. Pérez-Martín, M. López-Panadés, J. Urtasun-Elizari, P. Barbeito, I. Roig, F. R. Garcia-Gonzalo, J. Page, R. Gómez

**Affiliations:** 1 Unidad de Biología Celular, Departamento de Biología, Universidad Autónoma de Madrid, Madrid, Spain; 2 MRC Laboratory of Medical Sciences, London, United Kingdom; 3 Centro de Investigación Biomédica en Red en Enfermedades Cardiovasculares (CIBERCV), Madrid, Spain; 4 Vascular Research Laboratory, IIS-Fundación Jiménez Díaz, Madrid, Spain; 5 Department of Pharmacology, School of Medicine, Instituto de Investigación Sanitaria Gregorio Marañón, Universidad Complutense, Madrid, Spain; 6 Genome Integrity and Instability Group, Institut de Biotecnologia i Biomedicina, Universitat Autònoma de Barcelona, Cerdanyola del Vallès, Spain; 7 Cytology and Histology Unit, Department of Cell Biology, Physiology and Immunology, Universitat Autònoma de Barcelona, Cerdanyola del Vallès, Spain; 8 Instituto de Investigaciones Biomédicas Sols-Morreale (IIBM), UAM-CSIC, Madrid, Spain; 9 Departamento de Bioquímica, Facultad de Medicina, Universidad Autónoma de Madrid, Madrid, Spain; 10 CIBER de Enfermedades Raras (CIBERER), Instituto de Salud Carlos III, Madrid, Spain

**Keywords:** centrosome, cilia, cysts, meiosis, prepuberal mice, testis

## Abstract

Primary cilia have recently been identified in mammalian spermatocytes, but their developmental regulation during testicular maturation remains poorly understood. Here, we investigate the dynamics of ciliogenesis during mouse puberty and show that primary cilia are not an intrinsic feature of spermatocytes during the first wave of meiosis, initiated at 8 days post partum (dpp). Ciliogenesis begins only at day 20 dpp, where cilia are detected across all stages of prophase I, indicating no direct association with synapsis or desynapsis. We further found that Aurora kinase A is associated with cilia disassembly in late diplotene and that spermatocytes retaining a polymerized cilium fail to assemble a bipolar spindle. In addition, proteomic analysis defined the 19–21 dpp period as a key developmental window associated with ciliogenesis and flagellogenesis. We also identified ciliated spermatocytes in the human testis, providing the first evidence of meiotic cilia in human meiocytes. This work reveals that ciliogenesis is a developmentally regulated process during testicular maturation in mouse.

## Introduction

Cilia and flagella are microtubule (MT)-based plasma membrane projections that function as motile or sensory structures. Motile cilia and flagella have a well-established structure, typically exhibiting an axoneme with a 9 + 2 arrangement, with nine peripheral MT doublets connected to each other by dynein arms, and a central MT pair bound to peripheral doublets by radial spokes. This organization, along with the association of motor proteins, enables motile cilia and flagella to generate rhythmic movements that result in cell locomotion, extracellular fluid displacement or spermatozoan propulsion (Bornens, 2021; Bloodgood, 2010; Gadadhar et al., 2022). In contrast, primary cilia serve as signalling organelles, detecting mechanical, optical, or chemical stimuli (Derderian et al., 2023). These primary cilia exhibit unique features. First, they are solitary, with only one present per cell. Second, they are transient structures, only present during the G1/G0 phases of the somatic cell cycle. Third, they possess a 9 + 0 axoneme configuration. And finally, they lack motility since their axoneme does not contain motor proteins (Kumar and Reiter, 2021).

Despite functional and structural differences, motile cilia, primary cilia, and flagella share numerous molecular and architectural components. In all cases, the axoneme arises from the mother centriole of a membrane-docked centrosome, also referred to as the basal body. The mother centriole presents distal and subdistal appendages that facilitate the docking to the membrane (Nigg et al., 2014). Additionally, they share several components, such as MTs with acetylated tubulin (AcTub) or the ADP Ribosylation Factor Like GTPase 13B (ARL13B) (Garcia-Gonzalo and Reiter, 2012; Satir and Christensen, 2008; Joukov and De Nicolo, 2019). Notably, the assembly, maintenance, and functions of both motile and nonmotile cilia strictly depend on intraflagellar transport (IFT) proteins (Lacey et al., 2023; Wingfield et al., 2018). As cilia lack the translational machinery for protein synthesis, IFT serves as the sole mechanism for trafficking the essential components into the ciliary compartment required for cilia assembly, growth and functions.

Over the past century, research has uncovered the diverse functions of primary cilia in mammalian tissues, including roles in mechanosensing, vision, and chemical signal transduction. Playing these functions, cilia can detect a wide range of stimuli—including blood flow, light, hormones, and growth factors—underscoring their importance in cellular signalling (Derderian et al., 2023; Singla and Reiter, 2006; Chinipardaz et al., 2022; Anvarian et al., 2019). Given these roles, disruptions in ciliary structure or signalling pathways due to mutations in cilia-related genes result in a spectrum of diseases known as ciliopathies (Hildebrandt et al., 2011). These disorders can affect ciliary assembly and maintenance or, in the case of primary cilia, disrupt intracellular signalling pathways (Anvarian et al., 2019; Andreu-Cervera et al., 2021; Reiter and Leroux, 2017).

Advancements in our understanding of primary cilia have uncovered that ciliary assembly (ciliogenesis) and disassembly are dynamically regulated in coordination with the cell cycle (Wheatley et al., 1996). In somatic cells, primary cilia form in G1/G0 and are reabsorbed in G2, thereby freeing centrioles and enabling them to function as Microtubule Organizing Centers (MTOCs) at the spindle poles during cell division (Graser et al., 2007; Vertii et al., 2016; Kobayashi and Dynlacht, 2011). Key regulators of primary cilium disassembly appear to include many well-known master controllers of mitotic progression (Sánchez and Dynlacht, 2016; Plotnikova et al., 2009). Among these, the mitotic Aurora A kinase (AURKA) (Lukasiewicz and Lingle, 2009) has emerged as a crucial regulatory factor for somatic primary cilium disassembly. Activation of AURKA in the context of primary cilium resorption is mediated by a mechanism involving the phosphorylation and activation of deacetylase HDAC6 (Pugacheva et al., 2007; Nishimura et al., 2021).

Gametogenesis is a fundamental biological process responsible for generating gametes (oocytes and spermatozoa). Spermatogenesis, in particular, comprises at least three functional processes: i) proliferation of spermatogonia (stem germ cells), ii) meiosis, a specialized cell division that generates haploid cells (spermatids) from the diploid spermatogonia, and iii) spermiogenesis, i.e., the morphological maturation of spermatids into mature spermatozoa, which includes the formation of the flagellum (Griswold, 2016). In mammals, spermatogenesis occurs in the seminiferous epithelium of the testis, a highly specialized environment that allows nonsynchronous and continuous spermatogenesis. A key participant of spermatogenesis are the Sertoli cells, which are the supporting cells of the seminiferous epithelium and play a crucial role in providing the structural and hormonal environment for spermatogenesis (Griswold, 2018). Another key feature of the seminiferous epithelium is the fact that spermatocytes and spermatids are interconnected via cytoplasmic bridges, forming large cysts (Dym and Fawcett, 1971), which facilitates the biochemical and physiological coordination of cells within the epithelium (Greenbaum et al., 2011; Haglund et al., 2011; Sorkin et al., 2025).

The testis undergoes profound physiological and morphological transformations from fetal development to adulthood, driven by complex hormonal and molecular adjustments that promote the maturation of spermatogonia and the initiation of spermatogenesis (Rey et al., 2009; Nikolic et al., 2016; Shima et al., 2004). Remarkably, the testis is one of the few organs that establishes most of its cell types, functions, and physiology after birth (Geyer et al., 2017). Puberty marks a critical period during which the inaugural wave of complete spermatogenesis initiates with spermatogonial differentiation and culminates in the production of the first spermatozoa. This initial wave not only establishes male fertility but also sets the foundation for continuous sperm production throughout the reproductive lifespan. Hence, as the stage when fertility is established, puberty provides a critical window to investigate key events underlying spermatogenesis.

In recent years, primary cilia have been observed in several somatic cell types of the testis and epididymis, including Sertoli, peritubular myoid or epididymal cells (Nygaard et al., 2015). Moreover, the presence of cilia in germ cells has now been confirmed in various species, including *Drosophila melanogaster* spermatocytes (Riparbelli et al., 2012), zebrafish oocytes and spermatocytes (Mytlis et al., 2022; Xie et al., 2022), butterfly *Pieris brassicae* (Gottardo et al., 2023) and mouse spermatocytes (López-Jiménez et al., 2022). These findings challenge the long-standing paradigm that primary cilia must be disassembled prior to cell division. Indeed, the presence of the cilium seems to be critical for chromosome dynamics at the beginning of meiosis in zebrafish, and its disorganization can compromise meiosis outcome (Mytlis et al., 2022).

These findings open intriguing questions on the relationship between gametogenesis and ciliogenesis that call for a deeper characterization. Nevertheless, reports studying the extent and features of primary cilia in mammalian spermatocytes are scarce. In a previous study we found that in adult mice cilia are prominent and appear predominantly in zygotene-stage spermatocytes, suggesting that cilia can play an important role in the physiological and functional coordination of spermatocytes within the seminiferous tubules (López-Jiménez et al., 2022). In contrast, it has been suggested that in prepubertal mouse spermatocytes cilia may be much shorter or absent (Mytlis et al., 2022). This makes us suspect that there may be differences in the development and/or regulation of cilia formation in the first spermatogenesis wave compared to adults. To address this question, we have performed a systematic study of primary cilia morphogenesis in prepubertal mice, from day 8 until day 60 post-partum. Our results discovered that the timing of cilium formation in spermatocytes is temporally linked to flagellum development in spermatids. These findings suggest an unanticipated coordination in the formation of axonemal structures during the establishment of fertility in puberty, pointing to a broader developmental program governing ciliogenesis in the prepuberal male germline.

## Results

### The first wave of spermatogenesis is completed 28 days *post partum*

**FIGURE 1:**
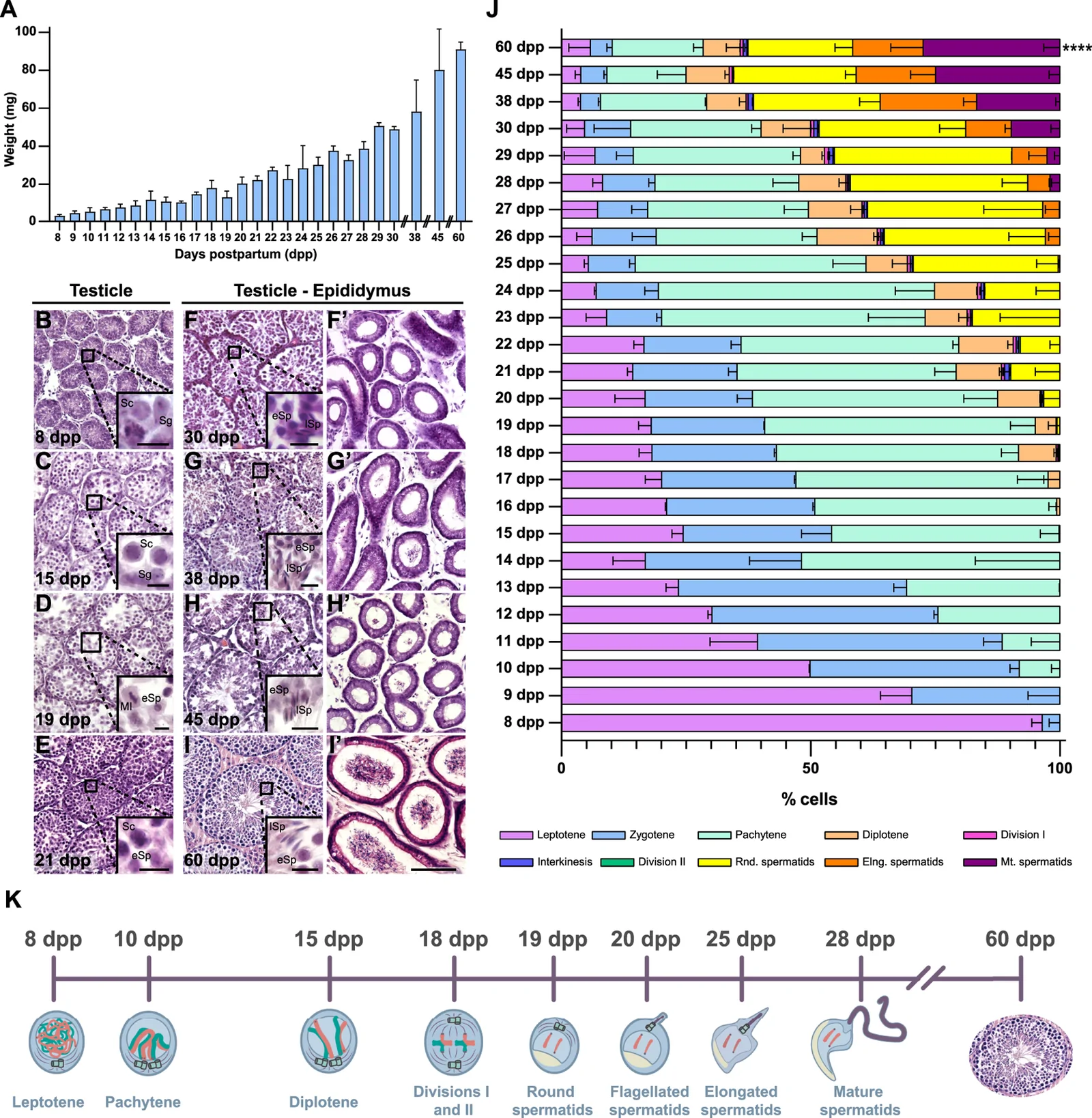
The first spermatogenesis wave in prepuberal mice. **(A)** Graphical representation of the weight of the testis in animals in the age range 8–60 days post partum (dpp) (two biological replicates from 8 to 22 dpp and three biological replicates from 23 to 60 dpp). (**B–I)** Cross sections of the H&E-stained testis or testis and epididymis **(F′–I′)** from 8, 15, 19, 21, 30, 38, 45 and 60 dpp mice. Scale bar represents 100 µm. Selected enhanced images of the different cell types are shown in bottom left squares. Sg: spermatogonia; Sc: spermatocyte; MI: metaphase I; eSp: early spermatid; lSp: late spermatid. Scale bar represents 10 µm. **(J)** Distribution of the meiosis stages in animals in the age range 8–60 dpp. The analysis quantifies stages at prophase I (leptotene, zygotene, pachytene and diplotene), division I (metaphase I, anaphase I and telophase I), interkinesis, division II (metaphase II, anaphase II and telophase II), and spermatids (round/immature, elongated, and mature). Data represents mean ± SD, a X^2^ test was made between 45 and 60 dpp, ****p < 0.0001 (two biological replicates for 8 and 9 dpp n = a minimum of 100 cells per individual, and two biological replicates for 10 to 60 dpp n = a minimum of 2000 cells per individual). **(K)** Schematic representation of the key events during the first spermatogenesis, indicating when a new cell population appears for the first time in the seminiferous tubules.

Given the discrepancies previously reported about ciliogenesis in mouse spermatocytes (Mytlis et al., 2022; López-Jiménez et al., 2022), we hypothesized about the possibility that ciliogenesis in spermatocytes is a progressive process occurring during puberty. We first characterized the progression of the first spermatogenesis wave in mice, as striking discrepancies in timeline were found across earlier studies (Nebel et al., 1961; Clermont, 1972; Bellve et al., 1977; Goetz et al., 1984; Shao et al., 2015). To this end, testes from prepubertal, pre-weaning C57BL/6 male mice were collected, measured, and weighed daily between 8 and 30 dpp (Figure 1), and we also analysed weaning males between 30 dpp and 60 dpp at weekly intervals. As expected, testis weight increased steadily with age, reaching adult-like weight between 45 and 60 dpp (Figure 1A). Stage 8 dpp marks the appearance of the first meiotic spermatocytes derived from self-renewing undifferentiated spermatogonia (Yoshida et al., 2006). At this age, the seminiferous tubules exhibited a small diameter and contained only spermatogonia and primary spermatocytes (Figure 1B). The diameter of the seminiferous tubules increased progressively, and round (immature) spermatids were first observed in testis sections at 19 dpp (Figures 1C–E). By 28-30 dpp, elongated spermatids and mature spermatids appeared in the lumen of the seminiferous tubules; however, these animals were still considered prepubertal, evidenced by the absence of mature spermatids or spermatozoa in epididymal sections (Figures 1F,F´). Fully mature spermatozoa were first detected in the epididymis at 38 dpp (Figures 1G,G´). From 45 dpp onwards, the testis was fully developed, with seminiferous tubules growing towards typical adult diameter and epididymal sections progressively filling with spermatozoa (Figures 1H,H´). At 60 dpp, epididymis is densely packed with spermatozoa (Figures 1I,I´).

We later characterized the proportion of spermatocytes at different stages during testis maturation (Figure 1J) by analysing the presence and localization of SYCP3 and SYCP1, proteins of the synaptonemal complex, along with γH2AX (a marker for DNA double-strand breaks, DSBs) in squashed seminiferous tubules of mice between 8 and 60 dpp (Supplementary Figure 1). We recorded the following stages: prophase I (leptotene, zygotene, pachytene, diplotene, diakinesis), first meiotic division (metaphase I, anaphase I and telophase I), interkinesis, second meiotic division (metaphase II, anaphase II and telophase II), and spermatids at different stages of spermiogenesis (round spermatids, elongated spermatids and mature spermatids). The most relevant findings indicate that at 8 dpp, 96.49% of spermatocytes are at the leptotene stage. Pachytene spermatocytes first appeared at 10 dpp, while the first, interkinesis and second meiotic divisions were firstly observed at 18 dpp (Figure 1J). Round spermatids were detectable from 19 dpp, with fully mature flagellated late spermatids first appearing at 28 dpp-30 dpp. The proportion of mature spermatids increased progressively between 28 and 45 dpp. Based on our data, 45 dpp can be considered the onset of adult testicular function. From 45 to 60 dpp, successive waves of complete spermatogenesis progressively increase the diameter of the seminiferous tubules, accompanied by a marked rise in the number of late spermatids. All values for Figure 1 are included in Supplementary Table 1. A summary graphic is included to illustrate the key stages and timing of the first spermatogenic wave (Figure 1K).

### Ciliogenesis simultaneously emerges across all stages of prophase I spermatocytes at 20 dpp

**FIGURE 2:**
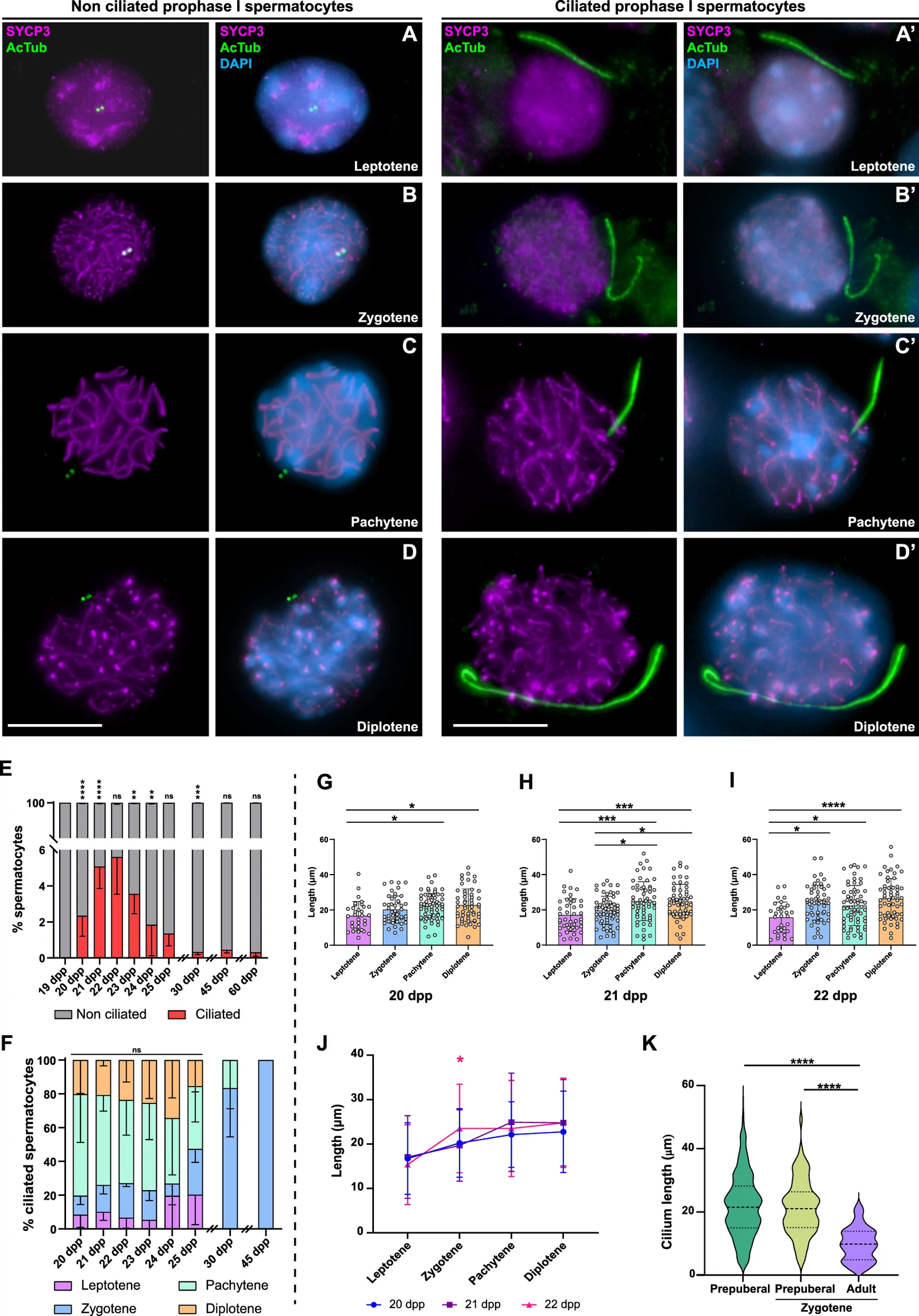
Cilia emerge across all stages of prophase I in spermatocytes during the first spermatogenesis wave. **(A–D)** Cilia appear in spermatocytes at leptotene, zygotene, pachytene and diplotene. Double immunolabelling of acetylated Tubulin (AcTub) (green) and synaptonemal complex protein 3 (SYCP3) (magenta) with chromatin stained with DAPI (blue) on squashed WT mouse spermatocytes at **(A,A′)** Leptotene, **(B,B′)** Zygotene, **(C,C′)** Pachytene and **(D,D′)** Diplotene. All images are z-projection of 30–60 frames of stack images of each cell. Scale bars represent 10 μm. **(E,F)** Cilia quantification. **(E)** Graphical representation of the quantification of ciliated spermatocytes at ages between 19 and 60 days post partum (dpp). Data represents mean ± SD, Χ^2^ test has been made between each date and the previous one, ns = no statistical significance, **p < 0.01, ***p < 0.001, ****p < 0.0001 (three biological replicates except for 45 dpp in which two biological replicates has been analyzed, n = a minimum of 500 spermatocytes per individual). **(F)** Graphical representation of the distribution of ciliated spermatocytes between different stages of prophase I for the age frame 20–45 dpp. Data represents mean ± SD, Χ^2^ test has been made between each date and the previous one, ns = no statistical significance (three biological replicates except for 45 dpp in which two biological replicates has been analyzed; for 20 dpp n = 54 ciliated spermatocytes; for 21 dpp n = 126 ciliated spermatocytes; for 22 dpp n = 95 ciliated spermatocytes; for 23 dpp n = 66 ciliated spermatocytes; for 24 dpp n = 34 ciliated spermatocytes; for 25 dpp n = 24 ciliated spermatocytes; for 30 dpp n = 5 ciliated spermatocytes; for 45 dpp n = 5 ciliated spermatocytes). **(G–K)** Cilia length quantification. **(G–I)** Graphical representation of cilia length at different stages of prophase I in **(G)** 20dpp, **(H)** 21 dpp and (I) 22 dpp prepuberal mice. Data represents mean ± SD, One-way ANOVA, *p < 0.05, ***p < 0.001, ****p < 0.0001 (three biological replicates, for 20 dpp n = 33 in leptotene, n = 46 in zygotene, n = 61 in pachytene, n = 53 in diplotene; for 21 dpp n = 43 in leptotene n = 62 in zygotene, n = 60 in pachytene, n = 55 in diplotene; for 22 dpp n = 31 in leptotene, n = 56 in zygotene, n = 62 in pachytene, n = 59 in diplotene). **(J)** Graphical representation of cilia length at different stages of prophase I in a combined analysis for 20–22 dpp mice. Data represents mean ± SD, One-way ANOVA, *p < 0.05 between 21 and 22 dpp, (three biological replicates, for 20 dpp n = 33 in leptotene, n = 46 in zygotene, n = 61 in pachytene, n = 53 in diplotene; for 21 dpp n = 43 in leptotene n = 62 in zygotene, n = 60 in pachytene, n = 55 in diplotene; for 22 dpp n = 31 in leptotene, n = 56 in zygotene, n = 62 in pachytene, n = 59 in diplotene). **(K)** Graphical representation of the global cilia length in all stages of prophase I spermatocytes, and specifically in zygotene in prepuberal versus zygotene spermatocytes in adult mice. Data represents mean ± SD, Kruskal-Wallis test, ****p < 0.0001. Prepuberal data (dark green) is the result of combining the data from 20 to 22 dpp (graphics G-I) (three biological replicates for each age, for prepuberal n = 621, and for zygotene stage in prepuberal n = 164) (three biological replicates for zygotene stage in adults n = 64).

We then aimed to identify the presence of cilia during prepuberal spermatogenesis. For this, we double immunolocalized SYCP3 and Acetylated Tubulin (AcTub), a marker of somatic (Piperno and Fuller, 1985) and meiotic (López-Jiménez et al., 2022) primary cilium. Results show that mice between 8 and 19 dpp did not present primary cilium in any spermatocyte. AcTub labeling was only detected at the centrosomes, as two small and rounded signals close to each other and to the cell nucleus, corresponding to the pair of centrioles (Figures 2A–D). In contrast, prepuberal individuals from 20 dpp onwards presented a small but significant proportion of spermatocytes at prophase I showing a ciliary axoneme labelled with AcTub (Figures 2A´–D´). An additional marker for cilia, polyglutamylated tubulin (GT335) was also assessed in ciliated and non ciliated spermatocytes (Supplementary Figures 2A–D,A`–D`).

Quantification analysis showed that ciliated spermatocytes at 20 dpp represented 2.34% of all prophase I cells, increasing to approximately 5% in mice at 21 and 22 dpp, and then progressively decreasing until 30–45 dpp, when the percentage of prophase I ciliated spermatocytes (0.43%) is similar to adulthood (Figure 2E), consistent with previously reported data for adult meiosis (López-Jiménez et al., 2022). Unexpectedly, cilia were observed at different stages of prophase I. This is in stark contrast to adult spermatogenesis, in which a primary cilium is only present at zygotene (López-Jiménez et al., 2022). Instead, in prepuberal mice, most ciliated spermatocytes were at pachytene stage, followed by diplotene and zygotene, while leptotene stage showed lower proportions of ciliated cells (Figure 2F). For clarity, we also include a graph showing the proportion of ciliated spermatocytes at each prophase I substage, calculated within the total number of cells in the same stage, for each of the ages of study (Supplementary Figure 2F). The sudden appearance of the primary cilium in different prophase I stages at 20 dpp, suggests that this is a simultaneous process in all prophase I spermatocytes at this developmental phase. Spermatocytes during later stages of the first and second meiotic division, or at interkinesis, did not present cilia.

Next, we measured the length of the primary cilium for each meiotic stage and age group. The length was found to vary depending on the meiotic stage, reaching its maximum at pachytene and diplotene stages. No differences were found in the length of the primary cilium between different ages, except between 21 and 22 dpp in zygotene cells (Figures 2G–J). Notably, prepuberal meiotic cilia are significantly longer (21.96 μm, rank: 3–55 μm) than adult cilia (10 μm) (López-Jiménez et al., 2022), even when comparing only ciliated spermatocytes at zygotene (Figure 2K).

### Cilia structure in mouse spermatocytes

The results presented above indicate that cilia are shaped differently in prepuberal compared to adult mice. To ascertain whether other features are shared or not, we analyzed additional markers for cilia and centrosome.

**FIGURE 3:**
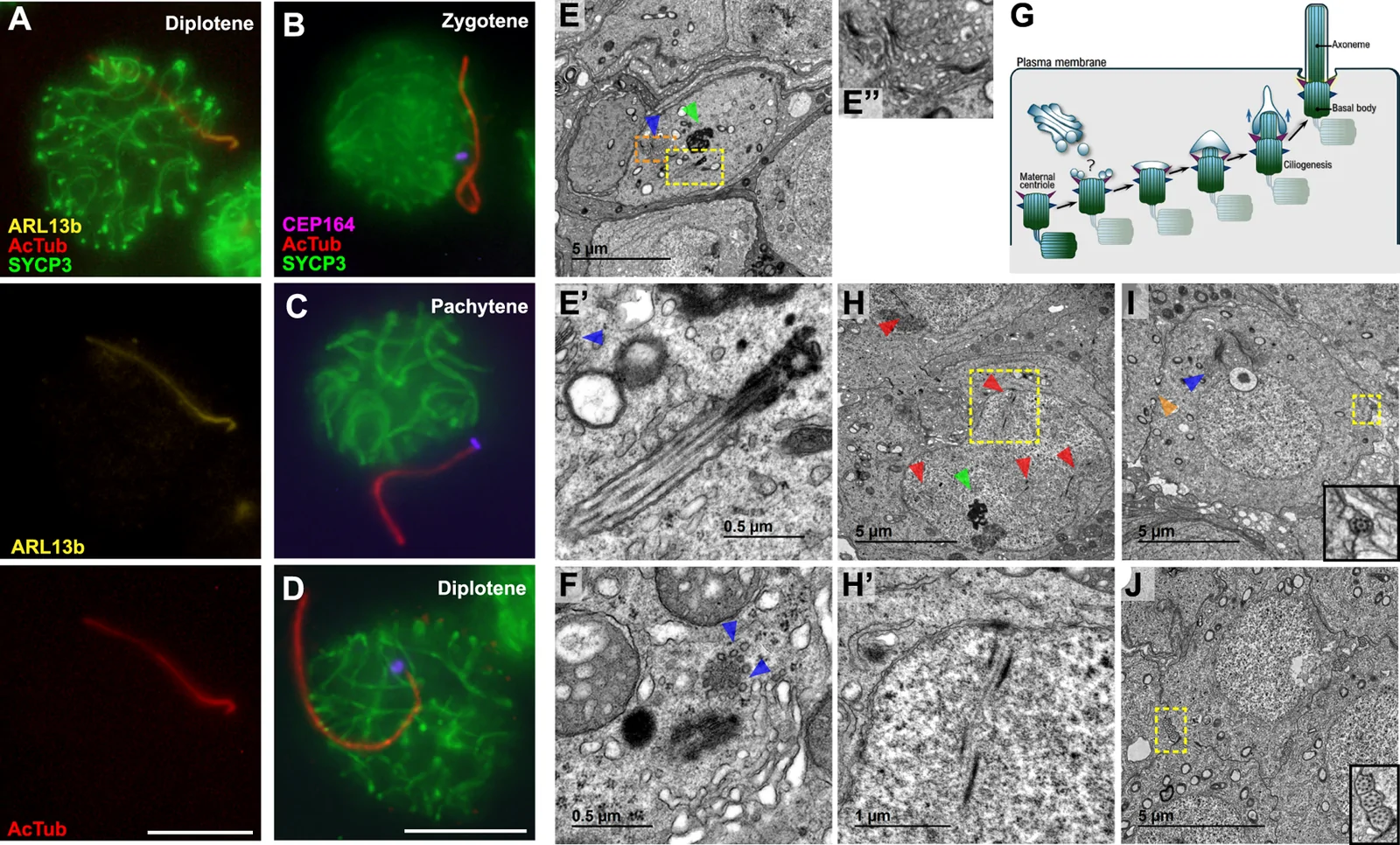
Ultrastructure and markers of prepuberal meiotic cilia. **(A)** Triple immunolabelling of acetylated Tubulin (AcTub) (red), synaptonemal complex protein 3 (SYCP3) (green) and ARL13b (yellow) in mouse spermatocytes in diplotene stage. (**B–D)** Triple immunolabelling of acetylated Tubulin (AcTub) (red), synaptonemal complex protein 3 (SYCP3) (green) and CEP164 (magenta) in mouse spermatocytes during prophase I (zygotene, pachytene and diplotene). All images are z-projection of 30–60 frames of stack images of each cell. Scale bars represent 10 μm. **(E–J)** Ultrastructure of axonemes in prepuberal meiosis. **(E)** Electron microscopy images of a ciliated spermatocyte. Amplified images of the cilia **(E′)** and the Golgi apparatus **(E´)**. **(F)** Centrosomes with Golgi derived vesicles in the maternal centriole. **(G)** Schematic representation of intracellular ciliogenesis pathway. **(H)** Prophase I spermatocyte. Amplified images of synaptnemal complex **(H′)**. **(I,J)** Mid spermatids presenting preacrosomic vesicle. Enhanced images of the flagella are offered. Blue arrowheads point to Golgi apparatus vesicles. Red arrowheads point to fragments of synaptonemal complex. Green arrowheads point to nucleolus. Orange arrowheads point to intercellular bridge between spermatids. Scale bars are included in each image.

Prepuberal cilia showed ARL13b and AcTub co-labelling (Figure 3A), as previously reported for adult ciliated spermatocytes (López-Jiménez et al., 2022). They arise from the maternal centrioles, labelled with CEP164 in all stages of prophase I (Figures 3B–D). To gain a more complete analysis of the prepuberal spermatocytes´s cilia, we further analyzed their ultrastructure under transmission electron microscopy (TEM) (Figures 3E–J). The meiotic cilia were observed in spermatocytes that present typical features of prophase-I, like a well-developed nucleolus and fragments of synaptonemal complex (Figures 3E,E´,H,H´; Supplementary Figure 3). Cilia axoneme emerges from one of the centrioles and typically lacks a central pair of MT. In contrast, flagella observed in early spermatids, already present in 21 dpp mice, are distinguishable for having a 9 + 2 axonemal organization and a clear preacrosomal vesicle near the nucleus (Figures 3I,J). We observed that meiotic cilia did not start to polymerize attached to the plasma membrane. Instead, they might emerge from the mother centriole of the centrosome, while it is still located internally in the cell, close to the nuclear envelope (Figure 3E). This suggests that cilia formation may follow an intracellular pathway, in which vesicles originating from the Golgi apparatus (Figure 3E’) are transported to the region above the maternal centriole, where they subsequently fuse (Figure 3F). Then, MT axonemes begin to polymerize within the cytoplasm and subsequently extend beyond the cell membrane into the extracellular space (see schematic diagram in Figure 3G). This pathway has been previously described for several somatic tissues (Hoffman and Prekeris, 2022; Rogowski et al., 2013; Sorokin, 1962; Martínez-Hernández et al., 2024). Taking advantage of the resolution of TEM, we have additionally analysed selected meiotic centrosomes at different stages of prophase I: at leptotene, prior to duplication, and at zygotene, after duplication (Supplementary Figures 4A–C). Centrosomes in spermatocytes at these stages have been rarely documented in recent decades, following the classic studies (Rattner, 1972).

**FIGURE 4:**
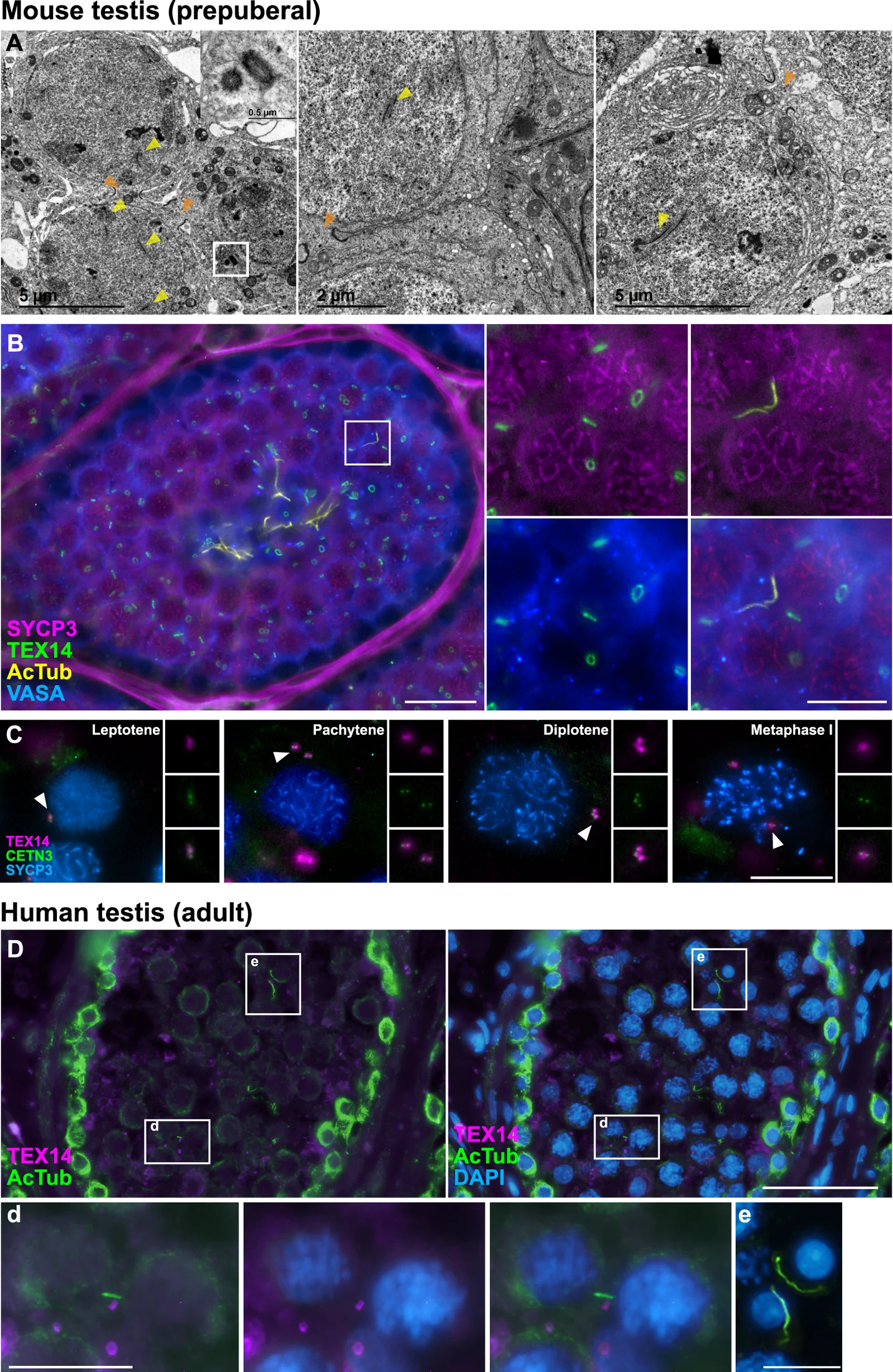
Testicular tissue presents spermatocyte cysts in prepuberal mice and adult humans. **(A,B)** Mouse spermatocytes are connected in cysts from the first round of spermatogenesis. **(A)** Electron microscopy images of spermatocytes at prophase I presenting fragments of synaptonemal complex (yellow arrowheads) which are connected by intercellular bridges (orange arrowheads). The centrosome of one of the spermatocytes is enhanced in an amplified image. Scale bars are included in each image. **(B)** Quadruple immunolabelling of synaptonemal complex protein 3 (SYCP3) (magenta), testis-expressed protein 14 (TEX14) (green), acetylated Tubulin (yellow) and the germ specific cytoplasmic marker VASA (blue) in testis cryosection of a 21 dpp mouse. Scale bars represent 20 μm for section images and 10 μm for amplified sections. **(C)** Triple immunolabelling of (TEX14) (magenta), Centrin 3 (CETN3) (green) and SYCP3 (blue) in squashed spermatocytes at prophase I (leptotene, pachytene and diplotene) and metaphase I. Scale bar represents 10 μm. **(D)** Human spermatocytes are connected in cysts and present cilia. Double immunolabelling of TEX14 (magenta) and acetylated Tubulin (green) in adult human testis cryosection. Chromatin is stained with DAPI (blue). Selected enhanced images correspond to a ciliated spermatocyte **(d)** and a flagellated early spermatid **(e)**. Scale bars represent 50 μm in A and 10 μm in **(d,e)**.

TEM analysis also showed that prophase I spermatocytes in prepuberal mice are interconnected via cytoplasmic bridges and share cytoplasmic components, including mitochondria (Figure 4A). These bridges, partially composed of the Testis-expressed protein 14 (TEX14), are present from the onset of the first meiotic wave and connect spermatocytes within the same cyst (Figure 4B), a feature also observed in adult mice (López-Jiménez et al., 2022). The continuity of the shared cytoplasm in cyst of spermatocytes is demonstrated in electron microscopy images (Figure 4A), and with the immunostaining of the germ specific cytoplasmic marker VASA (López-Jiménez et al., 2022; Azizi et al., 2020) (Figure 4B). In addition, TEX14 is detected in centrosomes, coinciding with CETN3 signals (Figure 4C). This histological organization is conserved in humans, where ciliated spermatocytes are similarly observed at the base of seminiferous tubules (Figure 4D). Additional yH2AX staining demonstrates the existence of prophase I ciliated human spermatocytes (Supplementary Figure 5). These findings indicate that cysts of interconnected spermatocytes are already established during the first meiotic wave persisting into adulthood in mouse, and at least in adulthood in humans. The presence of this histological organization in both mouse and human testes suggests that it may represent a conserved feature of mammalian spermatogenesis.

### Cilia and flagella appearance are temporarily linked during prepuberal meiosis

**FIGURE 5:**
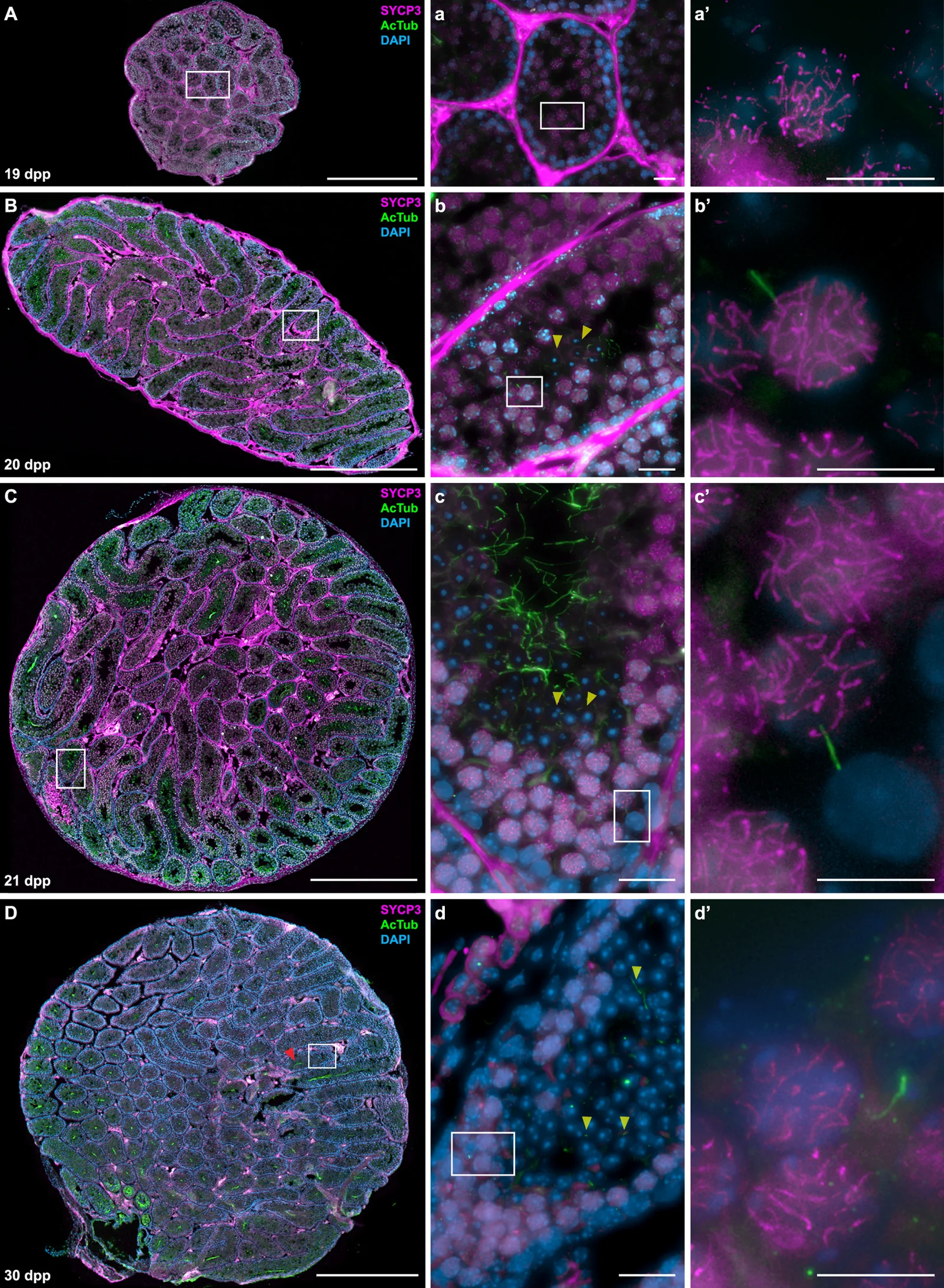
Cillia and flagella dynamics are correlated during prepuberal meiosis. **(A–D)** Double immunolabelling of acetylated Tubulin (green) and synaptonemal complex protein 3 (SYCP3) (magenta) in whole testis cryosection of 19 dpp **(A)**, 20 dpp **(B)**, 21 dpp **(C)** and 30 dpp **(D)** mice. Chromatin is stained with DAPI (blue). Enhanced images correspond to selected seminiferous tubules **(a–d)**, where ciliated spermatocytes are identified in the bases of the tubule after 20 dpp **(b´–d´)**. Early spermatids are indicated with yellow arrowheads, and late spermatids are indicated with red arrowheads. Scale bars represent 500 μm **(a–d)**, 20 μm **(a–d)** and 10 μm **(a´–d´)**.

As corroborated by microscopy analysis, the first appearance of cilia in spermatocytes occurs concurrently with the appearance of flagellated spermatids (Figures 1, 2). Thus, we asked whether these two events were occurring within the same seminiferous tubules. To keep the histological organization, we performed testis sections at 19, 20, 21 and 30 dpp, studying the distribution of AcTub in combination with SYCP3 (Figure 5). In agreement with our results in squashed spermatocytes, ciliated spermatocytes are not detected in 19 dpp mice, as only round and immature, yet still aflagellated, spermatids are present at this age (Figure 5A). The first ciliated spermatocytes and the earliest flagellated spermatids are observed at 20 dpp (Figure 5B). This histological organization is also observed in prepubertal mice at 21 dpp, when elongation of flagella had begun (Figure 5C), and at 30 dpp, were the percentage of ciliated prophase I spermatocytes is similar to adulthood (Figure 2E). By this stage, as shown in Figure 1, a lumen filled with mature spermatids resembling adult testis histology was observed (red arrowhead in Figure 5D).

These results indicate that the completion of the first meiotic wave at ∼20 dpp coincides temporally with the initial formation of axonemes in both cilia and flagella. This temporal correlation between ciliogenesis and flagellogenesis processes during testicular maturation highlights a potential framework for future mechanistic investigation.

### Proteomic analysis identifies potential regulatory factors of ciliogenesis and flagellogenesis

Given the critical relationship between gene expression and physiological functions, and the observation that ciliated spermatocytes emerge alongside flagellated spermatids at 20 dpp, we then aimed to identify potentially regulatory factors for axoneme formation during spermatogenesis. To achieve this, we conducted a comparative proteomic study using whole testicular extracts collected from multiple developmental stages: 8 dpp, 15 dpp, 19 dpp (before ciliogenesis), 21 dpp (after ciliogenesis), and adult. Although whole testes contain both germ and somatic cells, germ cells represent the predominant population during this developmental window and undergo major compositional changes between 15 dpp and adulthood, supporting the relevance of the detected proteomic shifts.

Proteomic analysis was performed using tandem mass tag (TMT)-based quantitative proteomics, a method that enables high-throughput, simultaneous quantification of thousands of proteins. Across all samples, a total of 4,304 proteins were detected (Supplementary Table 2). Quantitative data analysis was performed using PEAKS software, and all statistical analyses of the identified and quantified proteins were carried out in R for differential expression analysis. Differentially expressed proteins (DEPs) were defined as those showing a significant change in abundance between samples, with an adjusted p-value <0.05. A total of 621 DEPs were identified (Supplementary Table 3).

**FIGURE 6:**
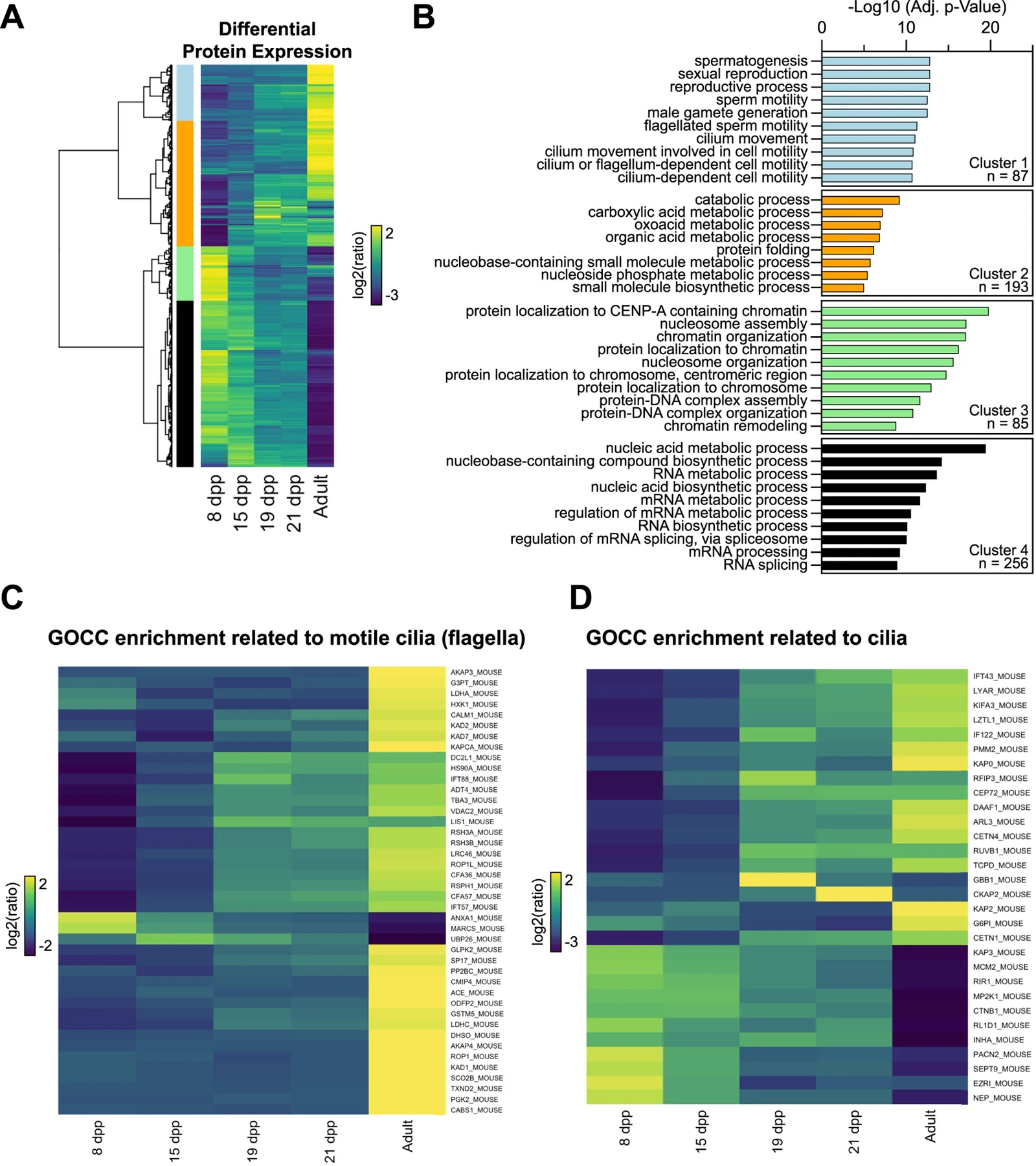
Comparative Proteomics Identifies Potential Regulators of Ciliogenesis and Flagellogenesis. **(A)** Cluster heatmap of the relative abundance of the differentially expressed proteins (DEP) in testis from 8 dpp, 15 dpp, 19 dpp, 21 dpp, and adult animals. Data was clustered based on K-mean analysis. Differentially expressed proteins (DEPs) were defined as those showing a significant change in abundance between samples, with an adjusted p-value <0.05. A total of 621 DEPs were identified and are shown in the heatmap. See Supplementary Table S2 for detailed data on each specific DEP. **(B)** Gene Ontology (GO) enrichment analysis in the different biological processes for the four differentially expressed proteins clusters. **(C,D)** Heatmap of dynamic abundances of the proteins associated with GO Cellular Component (GOCC) terms related to motile cilium (flagella) **(C)** and cilium **(D)**.

Using data from all ages studied, we generated a cluster heatmap of relative DEP abundance across development (Figures 6A,B). Gene Ontology (GO) analysis revealed biological processes enriched during testicular maturation. Four major DEP clusters were identified. Two clusters showed progressive increase towards adulthood, including proteins involved in sex reproduction and spermatozoa formation (cluster labelled in blue in Figure 6B) and those related to metabolic processes (cluster labelled in orange in Figure 6B). As expected, remarkable proteins that increase their expression towards adulthood are meiosis specific synaptonemal complex components SYCP3 and SYCE1 (Fraune et al., 2012). Other examples include lactate dehydrogenases A LDHA, and RNA/gene transcription regulators PIWIL1, FXR1, and chaperones such as HSP72, HSP90A, and calreticulin. These trends support the hypothesis that the testis undergoes a hypermetabolic phase as it transitions towards tissue maturation (Figure 6B; Supplementary Table 3). In contrast, two clusters showed decreased expression during maturation, corresponding to proteins involved in chromatin organization (green cluster in Figure 6B), and RNA processing (black cluster in Figure 6B), consistent with progressive cellular specialization.

To further explore cellular component associations (GOCC), we generated a heatmap of proteins related to axonemes of motile cilia, such the flagella in spermatids and spermatozoa (Figure 6C). Several flagellar proteins were upregulated during development. Notably, the testis specific form of LDHA, LDHC, peaked at 19 dpp, consistent with its role in flagellum formation (Goldberg et al., 2010). Additional proteins associated with axoneme assembly included TRiC complex components (TCPW, TCPE, TCPD, TCPA), which increased towards 19 dpp, coinciding with the emergence of ciliated spermatocytes and flagellated spermatids. This data is consistent with increased expression from 15 dpp onwards of Tubulin-Binding Protein TBB3, while TBA8 peaks expression almost exclusive to adulthood. This temporal correlation suggests a potential role for TRiC-mediated tubulin folding during axoneme assembly. Other enriched proteins from 19 dpp onwards include Intraflagellar transport proteins (IFT57, IFT88), dynein complex-related proteins DC2L1 (DYNC2LI1) (Hiyamizu et al., 2023), and Lis1 (Lissencephaly 1), which regulates microtubule dynamics during meiosis (Sitaram et al., 2012). Moreover, A-kinase anchoring proteins AKAP3 and AKAP4, localized in the flagellar fibrous sheath of the spermatozoa (Brown et al., 2003), also increased toward adulthood. Conversely, proteins such as ANXA1, involved in early germ cell differentiation (Huang et al., 2023), were more abundant at earlier developmental stages (8 and 15 dpp).

We next analyzed proteins associated specifically with primary cilia, by creating a specific GOCC heatmap of proteins linked exclusively to cilia but excluding those also found in motile cilia/flagella (Figure 6D). Several proteins showed increased expression during testis maturation. Notable examples related to primary cilia in other tissues include intraflagellar transport proteins IFT22 and IFT43 and the related traffic protein RAB11/FIP3 (Wang and Deretic, 2015), the GTPase ARL3 (with related ARL13b immunodetected at 20 dpp in Figure 3), centrosomal CETN1, CETN4, CEP72, and RUVBL1, became enriched at 19 dpp, preceding the appearance of primary cilia in spermatocytes. The detection of centriolar satellite protein CEP72, involved in the formation and function of primary cilia (Tyagi et al., 2025), and RUVBL1 (Mao et al., 2024), an ATPase involved in the cytoplasmic pre-assembly of ciliary protein complexes before their transport to the cilium (Mao et al., 2024), are interesting findings and empathize the novel value of 19–21 dpp developmental window for the study of axonemes during spermatogenesis. Conversely, other cilia-associated proteins, including Ezrin (Kawaguchi et al., 2022) and SEPT9 (Spiliotis and Nakos, 2021), showed reduced expression toward adulthood, suggesting roles in early ciliogenesis or roles in somatic testicular cell populations like undifferentiated Sertoli cells, in which cilia have also been previously reported (Nygaard et al., 2015).

Despite showing extensive changes in ciliogenic and flagellogenic proteins, our proteomic study did not reveal differences in components of Hedgehog (Hh) signaling, a cilia-dependent pathway known to be active in embryonic and adult testes (Bangs and Anderson, 2017; Bitgood et al., 1996; Sahin et al., 2014; Morales et al., 2009). To explore this further, we used immunoblot to quantify protein levels of GLI1, a transcription-dependent Hh pathway target, at different times during testicular maturation (8–60 dpp). Consistent with the proteomics, GLI1 protein levels remained stable during this period (Supplementary Figures 6A,B). Moreover, immunofluorescence of SMO, a signal transducer that accumulates in cilia upon Hh pathway activation, showed no ciliary staining in 21 dpp ciliated spermatocytes, while readily staining cilia in ASZ cells, a basal cell carcinoma line used as positive control (Choudhury et al., 2020; Bonilla et al., 2016) (Supplementary Figures 6C,D). Thus, our preliminary exploration suggests no major changes in transcription-dependent Hh signaling at these stages, but future studies will need to address this in more detail or explore other transcription-independent Hh pathways.

### Interference with meiotic cilia depolimerization causes meiotic defects

**FIGURE 7:**
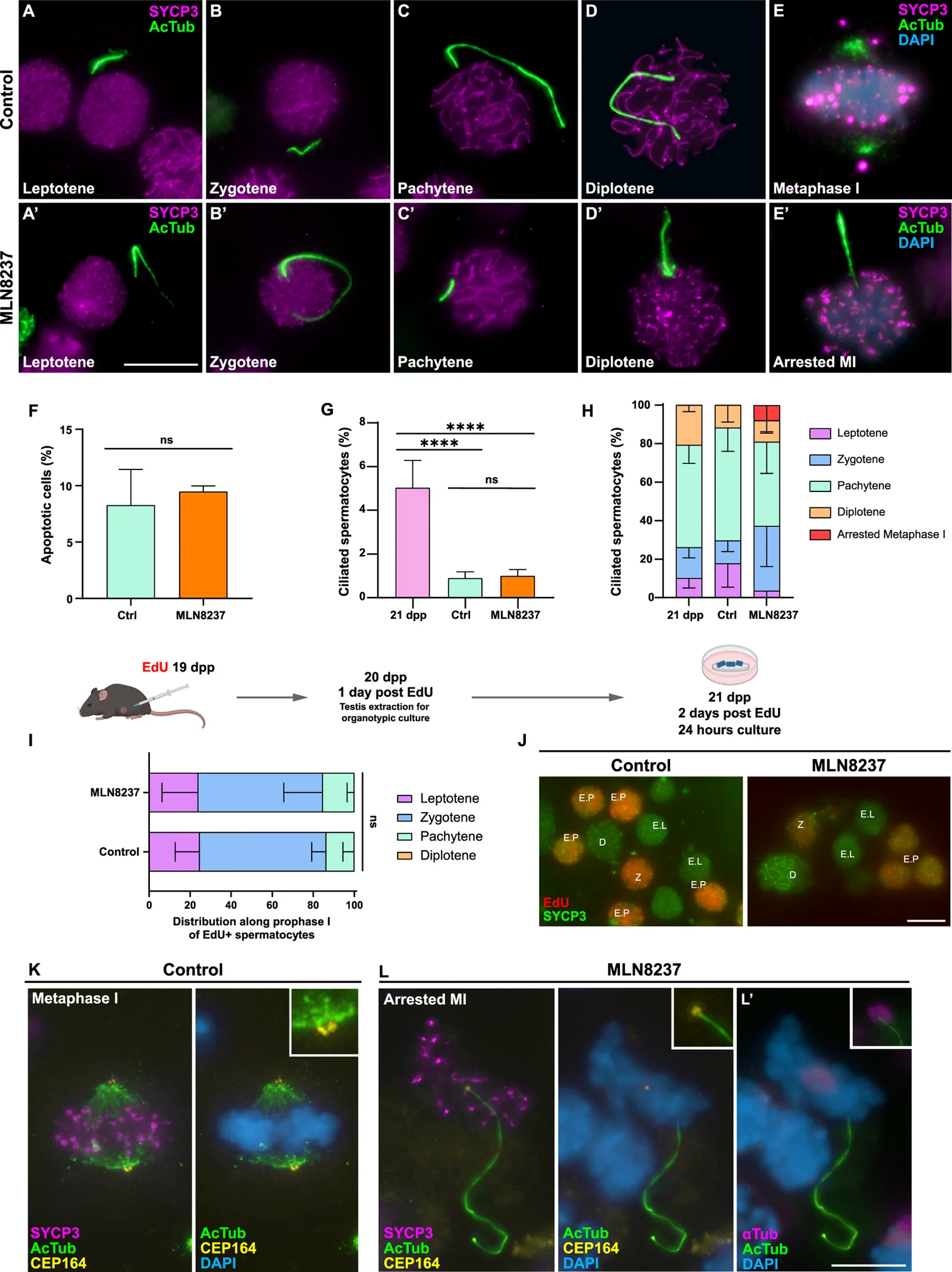
Aurora kinase A is a regulator of cilia disassembly in meiosis **(A–E)**. Aurora A inhibition causes interruption of cilia depolymerization. **(A–E)** Double immunolabelling of acetylated Tubulin (green) and synaptonemal complex protein 3 (SYCP3) (magenta) in cultured spermatocytes from 20 dpp mice. Chromatin is stained with DAPI (blue). Experiments were conducted in 24-h (24Hs) control **(A–E)** and 10 μM MLN8237 **(A′–E′)** cultured spermatocytes. Images are shown for squashed spermatocytes in all stages of prophase I (leptotene, zygotene, pachytene and diplotene) **(A–D)** and metaphase I (MI) **(E)** per each condition. All images are z-projection of 30–60 frames of stack images of each cell. Scale bar in A′ represents 10 μm. **(F–H)** Quantitative and qualitative analysis. **(F)** Graphical representation of the percentage of apoptotic spermatocytes in 24Hs control and MLN8237-treated cultured samples. Data represents mean ± SD, One-way ANOVA, ns = no statistical significance (three biological replicates, n = a minimum of 1500 spermatocytes per individual). **(G)** Graphical representation of the percentage of prophase I ciliated spermatocytes in fresh tissue from 21 dpp mice, compared to 24Hs cultured spermatocytes in control and MLN8237-treated samples. Data represents mean ± SD, Χ^2^ test, ns = no statistical significance, ****p < 0.0001 (three biological replicates, n = a minimum of 1500 spermatocytes per individual). **(H)** Graphical representation of the distribution of ciliated spermatocytes in fresh tissue from 21 dpp mice, compared to cultured tissue for 24Hs in control and CH-treated cultured samples. Data represents mean ± SD, (three biological replicates, for 21 dpp n = 126 ciliated spermatocytes; for control samples n = 60 ciliated spermatocytes; for MLN8237 treated n = 59 ciliated spermatocytes). **(I,J)** EdU pulse labelling indicates that Aurora A inhibition delays meiosis progression. **(I)** Graphical representation of the distribution of EdU-labelled spermatocytes among prophase I stages in control and 10 μM MLN8237 cultured samples. Data represents mean ± SD, Χ^2^ test, ns = no statistical significance (a minimum of 300 spermatocytes were quantified per condition, three biological replicates). **(J)** Image exemplification of cultured spermatocytes after EdU injection and organotypic culture. Double immunolabelling of EdU (red) and SYCP3 (green) in 24Hs control and MLN8237 cultured samples. E.L: early leptotene; Z: zygotene; EP: early pachytene; D: diplotene. Scale bar represents 10 μm. **(K,L)** The inhibition of cilia depolymerization during late prophase I induces the appearance of monopolar ciliated metaphases. **(K)** Triple immunolabelling of SYCP3 (magenta), acetylated Tubulin (green) and CEP164 (yellow) in control bipolar metaphases I. Chromatin is stained with DAPI (blue). **(L)** Triple immunolabelling of SYCP3 (magenta) or α-Tubulin **(L′)**, acetylated Tubulin (green) and CEP164 (yellow) in MLN8237-treated monopolar ciliated metaphases I. All images are z-projection of 30–60 frames of stack images of each cell. Scale bars represent 10 μm.

Our focus then shifted towards investigating whether the alteration of cilia disassembly may cause defects in the progression of meiosis. To investigate the consequences of blocking cilia disassembly, organotypic cultures of seminiferous tubules from 20 dpp mice were treated with the AURKA inhibitor MLN8237 (10 µM) for 24 or 48 h (Figure 7), following previously established protocols (Alfaro et al., 2021). We developed the experiment with cultured seminiferous tubules from 20 dpp mice, which already contain ciliated spermatocytes (Figure 2). This means that after a 24-h treatment, spermatocytes should behave as 21 dpp meiosis under Aurora A inhibition condition. Ciliated spermatocytes were observed in both control and MLN8237 24-h treated samples (Figures 7A–D,A′–D′). No significant increase in apoptosis was detected with cleaved caspase 3 after treatment, although the 48-h condition was excluded from further analyses because of progressive cell death (Figure 7F; Supplementary Figure 7).

Quantitative analysis revealed that MLN8237 treatment did not alter the total number of ciliated spermatocytes but significantly increased the proportion of ciliated zygotene spermatocytes (Figures 7G,H). To determine whether this reflected delayed meiotic progression or defective cilia disassembly, we performed EdU pulse-labelling experiments. The distribution of EdU-labelled leptotene, zygotene, and pachytene spermatocytes did not differ between fresh tissue, control cultures, and MLN8237-treated cultures (Supplementary Figure 8), confirming that EdU injection does not interfere with meiotic progression (Enguita-Marruedo et al., 2019). We then compared the distribution of EdU-labeled leptotene, zygotene, and pachytene spermatocytes between control and treated cultures 24-h post-culture and 48-h post-injection. To ensure temporal consistency, we only analyzed EdU-labeled spermatocytes that had entered prophase I at the same time. Results indicate AURKA inhibition did not significantly alter meiotic progression, yet MLN8237-treated cultures showed an increased proportion of ciliated zygotene spermatocytes (Figures 7H–J), indicating delayed cilia disassembly upon AURKA inhibition.

A striking finding of these experiments was the observation of ciliated spermatocytes at metaphase I. These ciliated spermatocytes were clearly anomalous, with aberrant SYCP3 signals at centromeres, abnormal chromatin compaction, and circular bivalent alignment with absence of AcTub- or α-Tubulin-positive spindle structures. This phenotype is consistent with AURKA inhibition in somatic cells disrupting cilia disorganization (Pugacheva et al., 2007), centrosome migration and spindle bipolarity (Alfaro et al., 2021; Wellard et al., 2021), therefore we classified them as arrested metaphase I spermatocytes (Figures 7E–E´,H). Both control bipolar spindles and MLN8237-induced monopolar spindles showed AcTub staining, but ciliated arrested metaphase I spermatocytes lacked any detectable spindle structure using α-Tubulin immunodetection (Figures 7E–E´,K,L,L´; Supplementary Figures 9A–C). This phenotype closely resembled the observed in PLK1 inhibition with BI2536 (Alfaro et al., 2021; Gomez et al., 2022), in which the AcTub-positive spindle is frequently absent in monopolar configurations (Supplementary Figure 9D). We further confirmed the loss of active AURKA, as indicated by the absence of auto-phosphorylated AURKA (detected by AURKTph antibody, as previously reported (Berenguer et al., 2022)) in monopolar spindles treated with a 24-h AURKA inhibition (Supplementary Figures 9E,F). Based on these observations, we conclude that the ciliated metaphase I spermatocytes in MLN8237-treated cultures possess an aberrant and arrested spindle. Further examination of CEP164, a marker of the mother centriole, revealed additional insights. In control metaphase I spermatocytes with bipolar spindles, four CEP164 signals were detected at opposite poles (Figure 7K), aligning with our previous observations as well as those reported by other authors (López-Jiménez et al., 2022; Wellard et al., 2021). However, in ciliated monopolar spindles from MLN8237-treated cells, CEP164 labeling was restricted to a single focus at the mother centriole, the one from which cilium emanates. This pattern also suggests impaired distal appendage formation and defective centrosome migration upon AURKA inhibition (Figure 7L; Supplementary Figures 9A–C).

Together, these findings indicate that cilia disassembly is required for centrosome maturation and bipolar spindle assembly during meiosis and identify AURKA as a critical regulator of these processes in spermatocytes.

## Discussion

This study provides, to our knowledge, the first evidence of ciliated spermatocytes across mammalian species, offering novel insight into germ cell biology. Previous reports using electron microscopy identified primary cilia in somatic testicular cells, including peritubular myoid cells in rabbits (Crabo, 1963), rats (Gaytan et al., 1986) and humans (De Kretser, 1967; Takayama, 1981), and more recent work reported cilia in immature Sertoli cells in the human testis (Nygaard et al., 2015), and fetal Leydig cells in mouse (Wainwright et al., 2014). However, these studies did not document primary cilia in meiotic germ cells. Our previous work was the first to report ciliated spermatocytes in adult mouse testes (López-Jiménez et al., 2022) and the present findings now extend this observation to prepuberal mice and provides evidence that meiotic ciliogenesis is also present in adult human spermatocytes, suggesting that this feature may be shared among mammals. Future studies in additional species will be required to determine its evolutionary conservation.

The evolutionary conservation of primary cilia in meiotic cells observed here supports the existence of a conserved structural feature whose functional significance remains to be determined. These observations provide a framework for future studies aimed at testing whether primary cilia contribute to processes such as meiotic progression, intercellular communication, or structural organization within germ cell cysts. Overall, these findings establish a descriptive foundation for exploring potential regulatory roles of primary cilia during testis development across mammals.

### Puberty: the foundation of fertility establishment in mouse

Previous studies reported discrepancies in the timing of the first meiotic wave during mouse testis development (Nebel et al., 1961; Clermont, 1972; Bellve et al., 1977; Goetz et al., 1984; Shao et al., 2015). Here, we refine this developmental timeline by identifying early spermatids in the seminiferous tubule lumen at 19 dpp, mature spermatids at 28 dpp, and full epididymal maturation at 60 dpp, consistent with previous reports (Gao et al., 2022).

**FIGURE 8:**
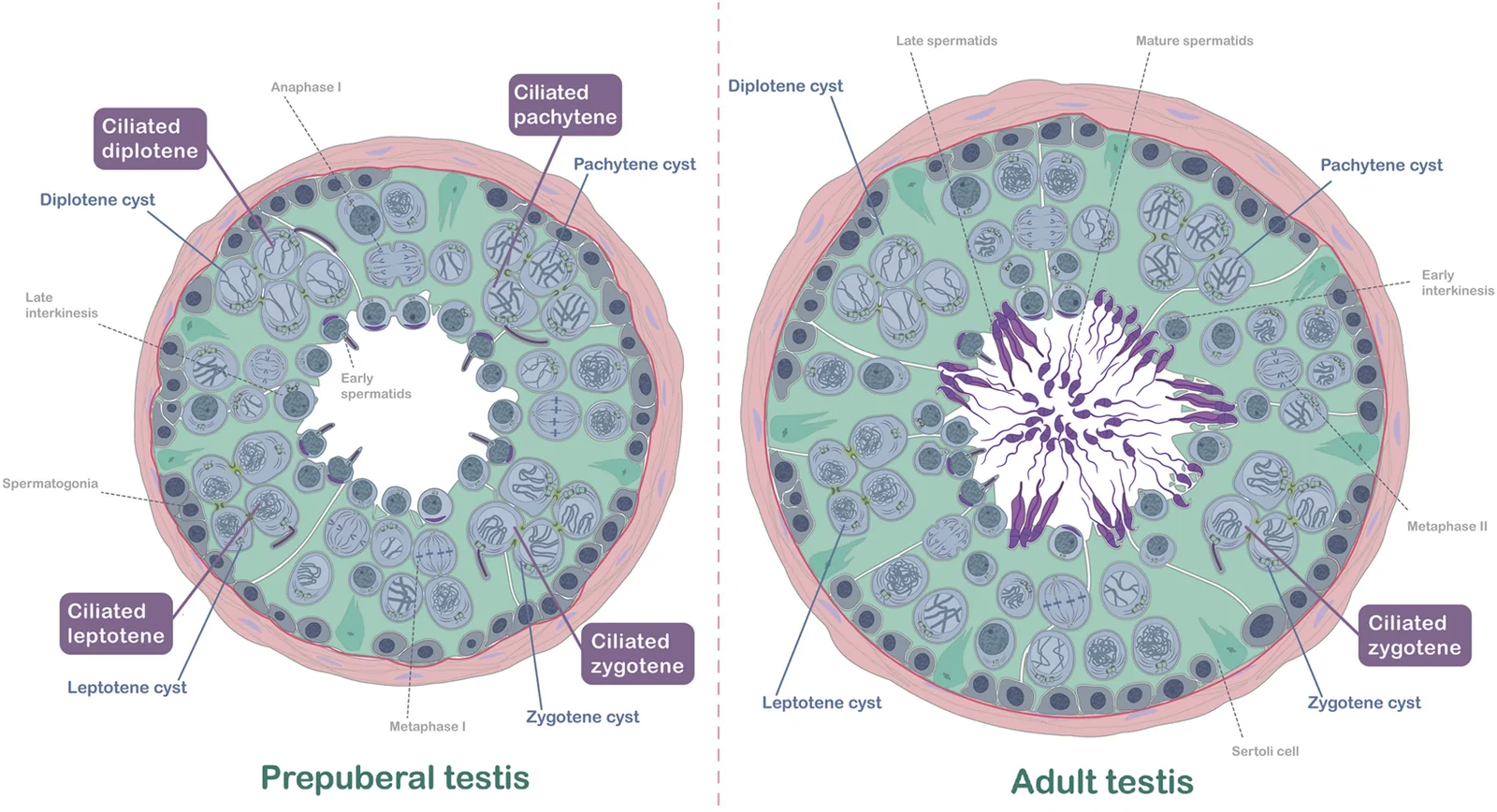
Schematic representation of the prepuberal versus adult seminiferous epithelium. Scheme represents a transversal section of a seminiferous tubule in testis of prepuberal and adult mouse. Spermatogonia (dark grey), spermatocytes (grey) and spermatids (purple) are embedded on the cytoplasm of Sertoli cells (turquoise). Centrosomes are indicated for every cell, in correspondence with timing of first centrosome duplication at zygotene, or second centrosome duplication at interkinesis. Cilia are represented in one of the prophase I spermatocytes of the cysts in prepuberal and adult mice. Intercellular bridges are indicated connecting the cytoplasms of the spermatocytes within the same cyst (highlighted in green). Myoid cells of the peritubular region are located surrounding the tubule (red).

Our work identifies meiotic ciliogenesis as a defining feature distinguishing prepubertal from adult spermatogenesis in mouse (Figure 8). We define the 19–20 dpp period as a pivotal developmental window during which ciliogenesis and flagellogenesis are synchronously initiated. From 20 dpp onwards, a small subset of spermatocytes exhibiting primary cilia are detected throughout all substages of prophase I, arguing against a direct association with synapsis or desynapsis. Cilia across all prophase I substages have also been reported in zebrafish spermatocytes (Xie et al., 2022), however, previous studies in zebrafish linked meiotic cilia to *bouquet* formation and synapsis initiation (Mytlis et al., 2022). In this same study, mouse spermatocytes from 12 to 16 dpp showed extremely short axonemal structures that barely extended the basal body (Mytlis et al., 2022). We found that ciliated spermatocytes appear only after 20 dpp, probably explaining why the previous study was unable to detect them.

Remarkably, spermatocyte cilia reached an average length of ∼22 μm, substantially longer than primary cilia in other mouse somatic tissues (e.g., 0.5–3 µm in brain, 1.5–3 µm in kidney or 5–7 µm in tracheal epithelium (Li et al., 2021)). Since long cilia have been associated with enhanced signaling capacity in other systems (Spasic and Jacobs, 2017; Macarelli et al., 2023), meiotic cilia may facilitate intercellular communication within cysts during testicular maturation.

The onset of ciliogeneis at 20 dpp temporally coincides with the appearance of immature spermatid flagella, suggesting coordinated developmental regulation without implying a direct causal relationship. Consistent with this interpretation, previous studies describing ciliated spermatocytes in the sterile *Ankrd31* ^−/−^ (Papanikos et al., 2019) indicate that cilia can assemble independently of flagellar formation in spermatocytes (López-Jiménez et al., 2022).

To further characterize the prepuberal to adult developmental transition in testis, we performed quantitative proteomic analyses across different ages, before and after the onset of meiotic ciliogenesis. Previous studies identified differential expression patterns during meiotic initiation at 8 and 10 dpp (Shao et al., 2015), or between 14 and 21 dpp (Huang and Sha, 2011), but proteomic data specifically focused on the 19–21 dpp window were lacking.

Interestingly, comparative proteomic analysis across the 8–60 dpp developmental window confirmed expected expression trends associated with testis maturation. Despite the relatively low abundance of ciliated spermatocytes, our analysis identified several candidate regulators associated with tubulin dynamics during testis development and defined the 19–21 dpp period as a molecular window associated with axonemal assembly, revealing increased expression of proteins linked to tubulin folding, ciliogenesis, intraflagellar transport, and flagellar organization. For instance, proteins such as LDHC, crucial for glycolytic metabolism (Odet et al., 2008) and PIWIL1, a key piRNA pathway component maintaining genome integrity (García-López et al., 2015), showed progressive increases toward adulthood. Most interestingly, heat shock proteins like HSP74L and HSP90A also showed robust upregulation, aligning with their established roles in maintaining spermatogenesis and sperm morphology (Dun et al., 2012; Kajiwara et al., 2012; Itoh et al., 2019). We also observed upregulation of tubulin folding chaperones, particularly TRiC complex components (TCPG, TCPE, TCPD, TCPA), especially from 19dpp onwards. The TRiC complex facilitates tubulin folding required for ciliary axoneme and flagellar assembly and has been implicated in ciliogenesis through interactions with the BBSome/IFT system (Nachury and Mick, 2019; Seo et al., 2010). Notably, the temporal increase of TRiC components coincides with the emergence of both cilia and flagella at ∼20 dpp in our immunostaining cellular and histological analysis, consistent with a potential contribution to axoneme assembly during this developmental window. Similarly, increased expression of tubulin-associated proteins such as TBB3 and the adult-specific tubulin isoform TBA8 supports their known roles in microtubule organization and flagellar assembly (Piprek et al., 2019; Gui et al., 2021; Sewell et al., 2024; Martianov et al., 2001; De Gendt et al., 2011). Likewise, DCAF7 required for male fertility (Ali et al., 2018) and linked to ciliogenesis through DYRK1A/B interactions (Kim and Kim, 2020; Tejedor and Hämmerle, 2011; Lee et al., 2022; Sacher et al., 2007), also showed elevated expression at this stage. Future mechanistic studies will be required to determine whether these interactions contribute directly to ciliogenesis or microtubule regulation during spermatogenesis.

We also detected cilia-associated proteins such as ARL3, RFIP3, RUVBL1, and several IFT components (IFT57, IFT88, IFT22, IFT43), with protein levels increasing during the same developmental window in which ciliated spermatocytes appear. The temporal concordance with cytological detection of ARL13B supports the developmental relevance of these observations. Finally, AKAP proteins (A-kinase anchor proteins), associated with flagellar structure and motility (Luconi et al., 2011), showed increased abundance toward adulthood, coinciding with the completion of spermatogenesis and the establishment of full fertility.

This proteomic dataset provides a developmental reference for the molecular events accompanying testis maturation, identifying candidate regulators associated with axoneme formation. Because the analyses were performed using whole-testis extracts, the cellular origin of the observed proteomic changes cannot be unequivocally assigned. Nevertheless, as germ cells undergo the most pronounced developmental changes during this period, the dataset provides a useful molecular framework for studying testis maturation and for identifying candidate regulators associated with ciliogenesis and flagellogenesis. These findings are consistent with recent studies using purified cell populations, including work by Fang et al. (2021), which identified cilium morphogenesis proteins in pachytene spermatocytes using purified cell populations, and are consistent with a coordinated relationship between ciliogenesis and testis development. Future studies using purified germ-cell populations will help define the cellular origin of the proteins identified in this dataset, whereas functional mechanistic analyses will be required to establish the roles of the candidate regulators identified here.

We further propose that meiotic cilia may function as signaling organelles coordinating communication between Sertoli cells and spermatocytes during puberty, consistent with the established role of primary cilia as sensory hubs for GPCR and RTK signaling pathways in somatic cells (Nachury and Mick, 2019; Goetz and Anderson, 2010). Our study focuses in the transition from the juvenile to the reproductive phase. It is therefore plausible that the transient presence of longer cilia during puberty reflects a developmental requirement for external signaling that becomes dispensable in the mature testis. To address this hypothesis, we explored whether these cilia might be involved in Hedgehog signaling, a pathway known to be functional in testes (Bangs and Anderson, 2017; Bitgood et al., 1996; Sahin et al., 2014; Morales et al., 2009). Our initial exploration suggests this is not the case, as indicated by: (i) the lack of Hh target proteins in our differential proteomics study; (ii) the unchanged protein levels of GLI1, a transcription-dependent Hh target, during testicular maturation; and (iii) the lack of SMO targeting to prepuberal spermatocyte cilia. Still, despite this initial evidence, more comprehensive future studies will be needed to solidly establish whether these cilia are or not involved in transcription-dependent or transcription-independent forms of Hh signaling.

Overall, our data provide, to our knowledge, the first integrated developmental molecular framework associated with axonemal structure formation and ciliogenesis during the 19–21 dpp developmental window. These findings establish a reference resource for future mechanistic studies, although functional validation will be required to define the specific roles of the candidate proteins identified here.

### Meiotic cilia share common regulators with somatic cells in mouse

Aurora kinase A (AURKA) is a master regulator of centrosomal functions and cilia resorption in somatic cells (Lukasiewicz and Lingle, 2009; Pugacheva et al., 2007; Nishimura et al., 2021; Asteriti et al., 2014; Tang et al., 2017). Microtubule remodelling, essential for centrosome migration, is modulated by the acetylation status of tubulin. Tubulin deacetylation enhances microtubule turnover and flexibility, facilitating reorganization required for spindle bipolarity. Conversely, tubulin acetylation stabilizes microtubules, reducing their propensity for depolymerization and impeding centrosome migration. In somatic cells, cytoplasmic deacetylases HDAC6 (Histone Deacetylase 6) (Hubbert et al., 2002; Zilberman et al., 2009) and Sirtuin 2 (Łysyganicz et al., 2021) mediate tubulin deacetylation, whereas α-tubulin acetyltransferase 1 (ATAT1) promotes microtubule stabilization and Polo-like-Kinase 1 (PLK1) recruitment to centrosomes (Rasamizafy et al., 2021). In addition, HDAC6 (Chawan et al., 2020) and ATAT1 had been pointed to play functional roles in mouse flagella (Yanai et al., 2021), although their interactions with AURKA during meiotic ciliogenesis remain to be determined. Since, PLK1 and AURKA (Alfaro et al., 2021; Wellard et al., 2021) localize to meiotic centrioles, our findings support the existence of a conserved regulatory centrosome-cilium crosstalk during male meiosis.

Our data suggest that AURKA regulates the transition from ciliated to deciliated states during late prophase I, adding to its previously described role in meiotic centrosome migration (Alfaro et al., 2021; Wellard et al., 2021). In somatic cells, cilia disassembly is required to release the basal body and allow spindle pole formation (Alieva and Vorobjev, 2004). By contrast, during meiosis, meiotic ciliogenesis begins once the first meiotic division is established - (López-Jiménez et al., 2022) and this study-, therefore the persistence of cilia may interfere with microtubule polymerisation and centrosome separation under AURKA inhibition, although these phenotypes may also reflect additional AURKA-dependent functions during meiosis. Our data suggest that AURKA drives cilia disassembly during late diplotene, and that spermatocytes retaining a polymerized cilium fail to form a bipolar spindle. These observations are consistent with the possibility that cilia maintenance and bipolar spindle organization are incompatible events during male meiosis. However, further validation using genetic approaches that specifically disrupt ciliogenesis will be required to test this hypothesis. This contrasts with observations in nonvertebrate organisms, like *Drosophila* and (Riparbelli et al., 2012) and butterfly *P. brassicae* (Gottardo et al., 2023), in which meiotic cilia persist through metaphase I without impairing bipolar spindle assembly.

Finally, the presence of ciliated spermatocytes both before and after centriole duplication at zygotene raises important questions about the interplay between ciliogenesis and centrosome duplication. In somatic cells, PLK4 maintains centriolar satellite integrity and promotes ciliogenesis through PCM phosphorylation (Hori et al., 2016), but is inactive during G1 to permit cilia formation (Bertolin and Tramier, 2020). In contrast, meiosis presents a unique scenario where PLK4 remains active throughout prophase I, driving centriole duplication (Skinner et al., 2024). The concurrent kinase activity of AURKA, PLK1 and PLK4 during male meiosis (Alfaro et al., 2021; Wellard et al., 2021; Skinner et al., 2024) suggests a potential crosstalk, possibly through PCM phosphorylation or other mechanisms, facilitating an integrated regulatory network coordinating centrosome function and ciliary dynamics. The presence of ciliated spermatocytes throughout all stages of prophase I raises the possibility that PLK4, PLK1 and AURKA may cooperate to regulate ciliary assembly or disassembly, either before or after centriole duplication. Future studies will be required to define the molecular interplay between ciliary proteins, centrosome regulators, and spindle dynamics during spermatogenesis.

In summary, this study establishes a descriptive and integrative framework for understanding meiotic ciliogenesis during mammalian testis development. By combining cytological, developmental, pharmacological and proteomic approaches, our findings define a previously unrecognized developmental window associated with axonemal assembly and highlight candidate regulatory pathways potentially linking ciliary dynamics to meiotic progression. Although further functional studies, including conditional genetic disruption of cilia-associated genes in germ cells, will be required to determine the precise role of primary cilia during male meiosis, this work provides a foundational resource for future studies of ciliogenesis in testicular maturation and fertility.

## Materials and Methods

### Materials

Testes from prepuberal and adult C57BL/6 (wild-type, WT) male mice were used for this study. Pre-weaning males were sampled at 8–30 days postpartum (dpp). Weaned males (not lactating from their mother) of 30–60 dpp were also used. All animal procedures were approved by local and regional ethics committees (UAM Ethics Committee for Research and Animal Welfare) and performed according to European Union guidelines.

Human samples were provided by Dr. Ignasi Roig (Universidad Autónoma de Barcelona, Spain). All human samples donation were approved by the corresponding institution (Hospital de Bellvitge, Barcelona) with the ethical committee reference BB20-024. Samples used for this study correspond to references B03-8544F y la B05-20957J of the correspondent biobank.

### Squashing methodology and immunofluorescence microscopy

Seminiferous tubules were fixed and processed following previously described protocols for the squashing technique (Page et al., 1998).

Acetylated Tubulin was detected with a mouse monoclonal antibody (Sigma, T7451) at a 1:1000 dilution. Polyglutamylated Tubulin was detected with a mouse monoclonal antibody (Adipogen, GT335) at a 1:500 dilution. SYCP3 was detected with a mouse monoclonal antibody (Santa Cruz, sc-74569) at a 1:200 dilution. SYCP1 was detected with a rabbit polyclonal antibody (Abcam, ab15090) at a 1:100 dilution. DSBs were detected with a rabbit polyclonal antibody against histone H2AX phosphorylated at serine 139 (γH2AX) (Abcam, ab2893) at 1:1000; CEP164 was detected with a rabbit polyclonal antibody (Sigma, ABE2621) at a 1:100 dilution; TEX14 was detected with a rabbit polyclonal antibody (Proteintech, 18351-1-AP) at a 1:500 dilution; VASA was detected with a rabbit polyclonal against mouse DDX4/MVH (Abcam, ab13840) at a 1:100 dilution; α-Tubulin was detected with a rat monoclonal antibody (Abcam, ab6160) at 1:100 dilution; histone H1t was detected with a guinea pig polyclonal antibody (kindly provided by Dr. Mary Ann Handel) at a 1:100 dilution. Pools of phosphorylated Aurora kinase A/B/C (AURKTph) were revealed with a rabbit monoclonal antibody anti-AuroraTph antibody (Cell Signaling, 2914S) at a 1:30 dilution. SMO was revealed with a rabbit polyclonal antibody (Abcam, ab.72130) at 1:400 dilution. Corresponding secondary antibodies were used against rat, rabbit and mouse IgGs conjugated with either AMCA, Alexa 488, Cy3 or Alexa 647 (Jackson Laboratories), all of them used at a 1:100 dilution.

Immunofluorescence images and stacks were collected on an Olympus BX61 microscope equipped with epifluorescence optics, a motorized Z-drive, and Olympus DP74 digital camera controlled by Cellsens software (Olympus Life Science). Finally, images were processed with ImageJ (National Institute of Health, USA; http://rsb.info.nih.gov/ij) or/and Adobe Photoshop softwares.

### Histology

For histological cryosections, dissected testis were fixated with 4% PFA for 24 h, then they were immersed in 15% and 30% sucrose solution 24 h for each step. Last, they were included in OCT (Sakura Finetek Europe) and immediately frozen. The OCT blocks were cut in 10 μm thick sections using a Leica CM1520 cryostat. Slides were washed in PBS three timesfollowed by permeabilization with PBS/0.1% Triton X-100 for 20 min. Then an antigen retrieval protocol was performed with sodium citrate 0,1M pH 6,0 at 85 °C for 30 min. After 3 washes in PBS, slides were blocked with PBS/2.5% BSA 0.1% Tween 20 for 30 min before being used for immunofluorescence protocol.

For human sections, samples were fixed in Formalin and included in paraffin. The paraffin blocks were cut in 7 μm thick sections. Then slides were dewaxed by washing three times in xylene, then in 100%, 96% and 75% ethanol two times per step, and finally in H_2_O. Then an antigen retrieval protocol was performed with sodium citrate 10mM, 0.5% Tween-20 pH 6,0 at 95 °C for 20 min. After 2 washes in PBS-T, slides were permeabilized with PBS/0.1% Triton X-100 for 20 min. Finally, before being used for immunofluorescence protocol, slides were blocked for 1 h using 0.3% BSA, 1% goat serum, glycine 0.00034M, 0.02% Triton X-100, 0.01% Tween-20 in PBS.

### Cell lines and culture and immunofluorescence microscopy

We used Basal Cell carcinomas (BCC) from the line ASZ, generously provided by Dr. Elisa Carrasco (Universidad Autónoma de Madrid), as positive control for the detection of active Hedgehog (Hh) pathway. For immunofluorescence protocol, cells were grown on coverslips were fixed in 3.7% formaldehyde/phosphate-buffered saline (PBS), permeabilized with 0.1% Triton X-100/PBS (1 h at room temperature, RT) and incubated for 1 h and 30 min at RT with the primary antibodies. Anti-acetylated tubulin (1:1000; mouse; Sigma T7451) and SMO (1:400; rabbit; Abcam, ab.72130). Corresponding secondary antibodies were used against rabbit and mouse IgGs conjugated with either Alexa 488 or 549 (Jackson Laboratories) at a 1:200 dilution, incubated for 1 h at RT. Finally, cells were counterstained with Hoechst for 5 min and mounted in ProLong (Invitrogen). Images were taken using an epifluorescence microscope linked to an Olympus DP50 digital camera.

### Electron microscopy

Testis from 21 dpp prepuberal mice were fixed in a mixture of 4% PFA, 2% glutaraldehyde in 0.1 M sodium cacodylate buffer, for 24 h. After fixing, samples were postfixed in a mixture of 1% osmium tetroxide and 0.8% potassium ferrocyanide, for an hour. Samples were treated with uranyl acetate 2% *en bloc* staining and dehydrated through a graded series of ethanol (30%, 50%, 70%, 95% and 100%), then embbebed in an acetone–Durcupan sequence and transferred to Durcupan epoxy resin. Ultrathin sections of 60 nm were obtained with Leica ultramicrotome and placed on grids. Finally, sections were stained with 2% uranyl acetate and Reynolds lead. For ultrastructural study, we used a Transmission Electron Microscope Jeol Jem 1010 (Tokyo, Japan) equipped with a Gatan Orius SC200 (Pleasenton, Ca) digital camera.

### Organotypic culture of seminiferous tubules and inhibition treatments

Culture of seminiferous tubules was performed as previously described (Alfaro et al., 2021; Sato et al., 2011), following previous reviewed recommendation (López-Jiménez et al., 2024). Testes from WT prepuberal mice were removed, detunicated and fragments of seminiferous tubules were cultured at 34 °C in an atmosphere with 5% CO_2_ onto agarose gel half-soaked in MEMα culture medium (Gibco A10490-01) supplemented with KnockOut Serum Replacement (KRS) (Gibco 10828-010) and antibiotics (Penicillin/Streptomycin; Biochrom AG, A2213). To inhibit AURKA, 10 µM of MLN8237 – also called Alisertib – (Selleckchem, S1133) diluted in 10% DMSO was added to the culture medium and seminiferous tubules were recovered after 24 or 48 h of treatment. Controls for each experiment were done with seminiferous tubules cultures in MEMα culture medium and 10% DMSO. After 24 or 48 h, control and inhibitor-treated seminiferous tubules were subjected to the squashing or spreading technique (described above). To inhibit PLK1, 100 µM of BI2536 was administrated during 24 h to organotypic culture of seminiferous tubules as previously reported (Alfaro et al., 2021).

### EdU pulse labelling

19 dpp prepuberal mice were injected intraperitoneally with 30 μL of 10 mM 5-ethynyl-2-deoxyuridine (EdU) (Thermo Fisher Scientific) diluted in PBS, and sacrificed after 24 h, 48 h, or 72 h, a timeframe that corresponds to 20 dpp, 21 dpp and 22 dpp. This allowed us to cover the ages when ciliated spermatocytes first appear at the testicular tissue tracking the first meiotic wave. Testicular samples were processed as described above and spermatocytes were labelled with anti-Acetilated Tubulin and anti-SYCP3. EdU was revealed using the Click-iT labelling as recommended by the supplier. For organotypic culture experiments, testis were extracted 24-h after EdU injection and cultured for 24 additional hours and processed as described in previous section.

### Quantification and statistical analysis

All values included in the figures are detailed in the Supplementary Table 1.

For the description of the first meiotic wave in prepuberal mice, 2000 spermatocytes were quantified in two biological replicates for each age (between 8 and 60 dpp).

For the quantitative analysis of the presence of ciliated spermatocytes, 500 spermatocytes in prophase I were analyzed in three biological replicates per condition (between 19 and 60 dpp). The length of cilia was quantified in a minimum of 30 spermatocytes per stage, for each analyzed age condition (20 dpp, 21 dpp or 22 dpp).

For the organotypic cultures experiments using MLN8237, 500 spermatocytes in prophase I were analyzed in three biological replicates per condition (control and MLN8237).

All statistical analyses were performed using GraphPad Prism 10 (GraphPad Software Inc.). For the analysis of differences in the number of cilia present a Χ^2^ test was performed. For the rest of the analyses, first, a normality test was performed for each analysis. Differences were analyzed by Mann-Whitney or one-way ANOVA when the data followed a normal distribution, and by a Kruskal-Wallis test when they did not. Then, a multiple comparison test was performed. Values are expressed as mean ± SD and P-values below 0.05 (P < 0.05) were considered statistically significant.

### Proteomic informatics analysis

The testes were decapsulated and homogenised with manual grinder in RIPA buffer (Sigma) supplemented with phosphatase and protease inhibitor cocktails. Following, the lysates were centrifuged at 12000 *g* for 15 min to remove insoluble material. Protein concentration was determined using the Pierce BCA Protein assay, and samples were separated by SDS-PAGE to assess protein quality. Subsequently, proteins were concentrated by running them on a stacking SDS-PAGE gel and digested using trypsin. The resulting tryptic peptides were desalted, concentrated and labelled with TMT 6-plex reagents following the manufacturer’s instructions. The efficiency of TMT labelling was evaluated through a preliminary LC-MS/MS analysis, and data quality was assessed accordingly. The TMT-mix was then fractionated using reverse-phase spin columns at basic pH. Each of the resulting fractions was analysed by LC-MS/MS on an LTQ-Orbitrap Velos Pro mass spectrometer using a short gradient method.

Quantitative data analysis was performed in PEAKS software and all statistical analysis of the identified and quantified proteins were performed in R statistical software (http://www.R-project.org/), DAVIS and GraphPad software (Prism 10, version 10.4.2). Tandem mass spectra were searched against the Swiss-Prot mouse database, applying a 1% false discovery rate (FDR) at both the peptide and protein levels. Proteins were considered confidently identified when supported by at least two unique peptides, with a protein-level FDR below 0.01. The relative abundance of each protein was defined as the log2 of the ratio between its abundance at a specific testis maturation stage and its mean abundance across all testis maturation stage Differential Expressed Proteins (DEPs) among the five stages of testis maturation were defined by two-way ANOVA filtered with an adjusted q value < 0.05. To identify DEP groups with similar abundance patterns during different stages of testis maturation, the optimal number of clusters (k value) was first determined using the Cluster package in R (version 4.3.3). Subsequently, relative abundance values of the DEPs were clustered using K-means analysis. DEPs were subjected to Gene Ontology (GO) enrichment analyses using the DAVID version v2024q4 (Sherman et al., 2022; Huang da et al., 2009). Functional annotation clustering was performed with default settings, and enriched GO terms were considered significant upon an adjusted p-value (Bonferroni corrected) less than 10e-5.

The categorization of protein as being involved in ciliogenesis or flagellogenesis was based on their Gene Ontology (GO) cellular component annotations obtained from the PANTHER database (Version 19.0), using the gene IDs of the Differentially Expressed Proteins (DEPs). Specifically, we used the GO terms cilium (GO:0005929) and motile cilium (GO:0031514). Since motile cilium is a subcategory of cilium, proteins annotated only with the general cilium term, but not included under motile cilium, were considered to be associated with primary cilia or with shared structural components common to different types of cilia.

### Western blot analysis and quantification

Lysates were then processed for SDS-PAGE and Western blot as previously described (Barbeito et al., 2023 Frontiers Cell Dev Biol 11: 1190258). Briefly, 30 µg of each lysate were loaded into 8% SDS-PAGE gels. After transfer to nitrocellulose, membranes were blocked and incubated with the following primary antibodies: mouse anti-GLI1 (Proteintech, 66905-1-Ig, 1:1000), or mouse anti-alpha-tubulin (Proteintech, 66031-1-Ig, 1:10,000). Membranes were then washed and incubated with these secondary antibodies: horseradish peroxidase (HRP)-conjugated goat anti-mouse IgG (Thermofisher, A16072) or HRP-conjugated rabbit anti-goat IgG (Thermofisher, #31402). After three more washes, signal detection was performed using SuperSignal West Pico PLUS Chemiluminescent Substrate (Thermofisher, 34578) and a Fusion Solo S imaging system (Vilber).

For band quantification, ImageJ software was used. Briefly, 8-bit grayscale images of blots were inverted, and average pixel intensity of each band was then obtained by multiplying average pixel intensity by pixel number. To this value, total pixel intensity of an equally sized background area was subtracted in order to obtain the specific band signal, which was expressed as a ratio to the control band.

## Data Availability

The datasets presented in this study can be found in online repositories. The names of the repository/repositories and accession number(s) can be found in the article/Supplementary Material.

## Supplementary material

SUPPLEMENTARY FIGURE 1 Meiotic stages during the first meiotic wave. **(A–G)** Double immunolabelling of synaptonemal complex protein 3 (SYCP3) (magenta) and synaptonemal complex protein 1 (SYCP1) (green). Images are shown for squashed spermatocytes in all stages of prophase I (leptotene, zygotene, pachytene and diplotene) **(A–D)**, metaphase I **(E)**, interkinesis **(F)** and metaphase II **(G)**. All images are z-projection of 30-60 frames of stack images of each cell. Chromatin is stained with DAPI (blue) in **(A′–G′)**. Scale bar represents 10 μm. **(H–L)** Double immunolabelling of synaptonemal complex protein 3 (SYCP3) (green) and DNA double-strand break marker γH2AX (red). Images are shown for squashed spermatocytes in all stages of prophase I (leptotene, zygotene, pachytene and diplotene) **(H–K)** and diakinesis **(L)**. All images are z-projection of 30-60 frames of stack images of each cell. Chromatin is stained with DAPI (blue) in **(H′–L′)**. Scale bar represents 10 μm.

SUPPLEMENTARY FIGURE 2 Non ciliated prophase I spermatocytes does not contain other primary cilia markers. **(A–D)** Triple immunolabelling of polyglutamylated Tubulin (green), acetylated Tubulin (AcTub) (red) and synaptonemal complex protein 3 (SYCP3) (magenta) with chromatin stained with DAPI (blue) on squashed WT 21 dpp mouse spermatocytes at **(A,A´)** Leptotene, (**B,B´)** Zygotene, **(C,C´)** Pachytene and **(D,D´)** Diplotene. All images are z-projection of 30-60 frames of stack images of each cell. Scale bars represent 10 μm. **(E)** Triple immunolabelling of ARL13b (green), acetylated Tubulin (red) and synaptonemal complex protein 3 (SYCP3) (magenta) on squashed WT 21 dpp mouse spermatocytes at pachytene. All images are z-projection of 30-60 frames of stack images of each cell. Scale bar represent 10 μm. **(F)** Graphical representation of the number of ciliated spermatocytes per each stage of prophase I for the age frame 20-45 dpp. Data represents mean ± SD, (three biological replicates, n=a minimum of 500 total spermatocytes per individual).

SUPPLEMENTARY FIGURE 3 Ultrastructure of prophase I spermatocytes. Electron microscopy images of spermatocytes at prophase I presenting fragments of synaptonemal complex (yellow arrowheads), and a prominent nucleolus (cyan arrowheads). The nuclear envelope (NE) is indicated.

SUPPLEMENTARY FIGURE 4 Meiotic centrosome under the electron microscope. **(A–C)** Electron microscopy images of spermatocytes in early prophase prior to centrosome duplications **(A,B)**, and after centriole duplication **(C)**. The nuclear envelope (NE) has been indicated as well as the synaptonemal complex (yellow arrowheads), nucleolus (cyan arrowheads) and intercellular bridges (orange arrowheads). Centrosomes are enhanced in the correspondent image **(A′–C′)**. The pair of centrioles **(C)** with their pro-centrioles (ProC) are clearly seen during centrosome duplication **(C′)**. Scale bars are included in each image.

SUPPLEMENTARY FIGURE 5 Human testis contains ciliated spermatocytes **(A–C)**. Immunolabelling of acetylated Tubulin (AcTub) (red) in adult human testis cryosections. Chromatin is stained with DAPI (blue). **(C)** Selected enhanced images correspond to two ciliated spermatocytes at the bases of the seminiferous tubule. Scale bars represent 50 μm in A, 20 μm in B and 10 μm in C. **(D,E)** Double immunolabelling of acetylated Tubulin (AcTub) (green) and DNA double-strand break marker γH2AX (magenta). Chromatin is stained with DAPI (blue). **(E)** Selected enhance image of a ciliated late prophase I spermatocytes. Scale bars in D and E are 10 μm.

SUPPLEMENTARY FIGURE 6 GLI1 readouts of Hedgehog signaling are not visibly affected in pubertal mouse testes. **(A)** Testis extracts from mice of various postnatal ages were resolved by SDS-PAGE and analyzed by Western blot using antibodies against GLI1 and α-tubulin as loading control. Molecular weight markers in kilodaltons are on the right. Mouse ages are indicated as postnatal days (8, 15, 19 and 21 dpp) or as adult (60dpp). Three biological replicates (n = 3) were analyzed for each age group. **(B)** Quantification of GLI1/tubulin band intensity ratio. The compared bands were in the same gel and blot. Data represent mean ± SEM, Tukey test, *p<0.05 compared to 8 dpp (three biological replicates). **(C,D)** Double Immunolabelling of Smo (magenta) and acetylated Tubulin (AcTub) (green) in prepuberal 21 dpp spermatocytes **(C)** and basal carcinoma cells **(D)**. All images are z-projection of 30-60 frames of stack images of each cell. Chromatin is stained with DAPI (blue). Scale bar represents 10 μm.

SUPPLEMENTARY FIGURE 7 Optimization of the MLN8237 protocol. **(A)** Double immunolabelling of synaptonemal complex protein 3 (SYCP3) (magenta) and cleaved caspase 3 (green) in 40x magnification fields of cultured squashed spermatocyes from 20 dpp mice. Chromatin is stained with DAPI (blue). Experiments were conducted in 24-hours (24Hs) and 48-hours (48Hs) in control and 10 μM MLN8237. Scale bar represents 20 μm. **(B)** Graphical representation of the percentage of apoptotic spermatocytes for each condition of spermatocyte culture. Data represents mean ± SD, One-way ANOVA, ns=no statistical significance (three biological replicates, n= a minimum of 5500 spermatocytes per condition). **(C)** Graphical representation of the percentage of prophase I ciliated spermatocytes for each condition of spermatocyte culture. Data represents mean ± SD, Χ^2^ test, ns=no statistical significance (three biological replicates, n= a minimum of 500 spermatocytes per individual).

SUPPLEMENTARY FIGURE 8 Tracking first meiosis wave with EdU pulse injection during prepubertal meiosis. Schematic representation of the protocol of EdU pulse. Injection of EdU is administered at 19 days post partum (dpp), followed by sacrifice of the animal an extraction of the testis after 24, 48 or 72 hours. **(A–C)** Double immunolabelling of synaptonemal complex acetylated tubulin (AcTub) (red) and EdU (magenta) or synaptonemal complex protein 3 (SYCP3) (green) in squashed spermatocytes at prophase I stages (leptotene, zygotene and pachytene). Ciliated spermatocytes are presented for each stayed being EdU or non-EdU labelled, depending on the time of testis extraction. Scale bars represent 10 μm. **(D–F)** Cryosection of the testis for timeframes 24, 48 or 72 hours after EdU injection. Triple immunolabeling of EdU labelling (red), synaptonemal complex protein 3 (SYCP3) (green) and acetylated tubulin (AcTub) (yellow). Chromatin is stained with DAPI (blue). Scale bars represent 50 μm. **(D′–F′)** A magnified image of ciliated spermatocytes located at the base of the seminiferous tubules are presented. Scale bars represent 10 μm. **(G,H)** Schematic representation of the protocol of EdU pulse and posterior organotypic culture of seminiferous tubules. Injection of EdU is administered at 19 days post partum (dpp), followed by sacrifice of the animal an extraction of the testis after 24 hours, and posterior culture of fragments of seminiferous tubules for additional 24 or 48 hours. **(G)** Graphical representation of the percentage of prophase I labelled with EdU after injection at 19 dpp and posterior incubation of spermatocytes during 24Hs in control and 10 μM MLN8237 samples. Data represents mean ± SD, Χ^2^ test (a minimum of 300 spermatocytes were quantified per condition, three biological replicates). **(H)** Double immunolabeling of EdU labelling (red) and SYCP3 (green) in 40x fields of prophase I spermatocytes for control and MLN8237 condition. Each is labelled with its corresponding stage (E.L: early leptotene, Z: zygotene, E.P: early pachytene; M.P.: mid pachytene; D. Diplotene). Notice that given the absence of blood flow in the culture, there is no new incorporation of EdU in early leptotenes that are initiating meiosis. Scale bars represent 10 μm.

SUPPLEMENTARY FIGURE 9 PLK1 and AURKA inhibition in cultured spermatocytes **(A–C)**. Double immunolabelling of acetylated Tubulin (AcTub) and α-Tubulin (red) in control **(A)** and treated with MLN8237 spermatocytes **(B,C)**. A monopolar non-ciliated metaphase I is shown in B and an arrested ciliated metaphase I showing no spindle is shown in C. Chromatin is stained with DAPI (blue). All images are z-projection of 30-60 frames of stack images of each cell. Scale bars represent 10 μm. **(D)** PLK1 inhibition with BI2536 following previous reports. Double immunolabelling of acetylated tubulin (AcTub) (red) and synaptonemal complex protein 3 (SYCP3) (green) in a monopolar metaphase I. Chromatin is stained with DAPI (blue). **(E)** Double immunolabelling of phosphorylated AURK (AURKTph) (red) and synaptonemal complex protein 3 (SYCP3) (green) in control bipolar metaphase I and metaphase II. Chromatin is stained with DAPI (blue) **(F)**. AURKA inhibition with MLN8237 following previous reports. Double immunolabelling of phosphorylated AURK (AURKTph) (red) and synaptonemal complex protein 3 (SYCP3) (green) in a monopolar metaphase I.
