# Supplementary material for: The dynamics of ciliogenesis in prepubertal mouse meiosis reveals new clues about testis maturation
Source: Front Cell Dev Biol. 2026 Aug 14;14:1910205. doi: 10.3389/fcell.2026.1910205 (PMC13522187; doi:10.3389/fcell.2026.1910205)
Supplement: Supplementary file 1 [file Image3.pdf]

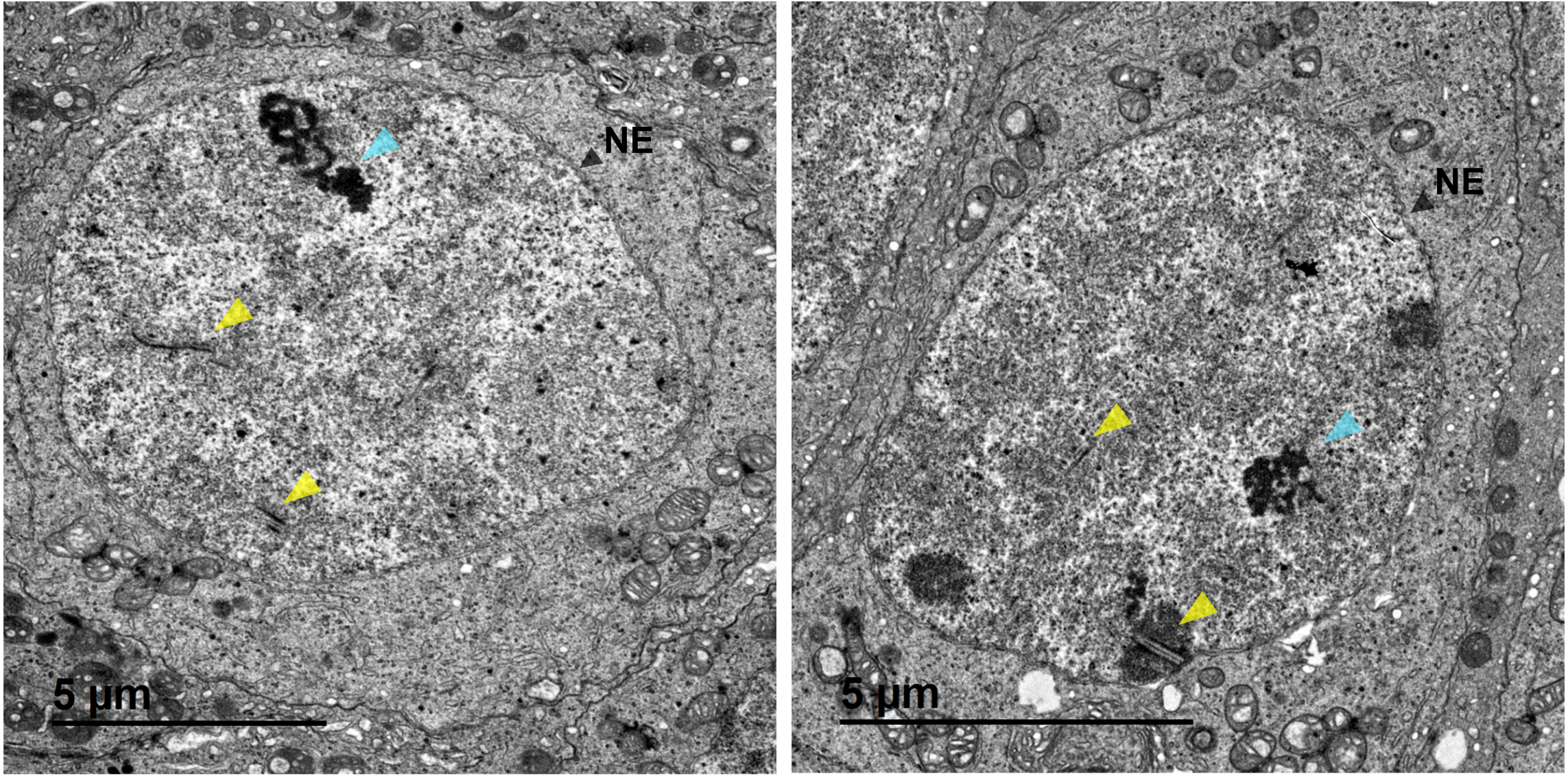
NE
5 µm
NE
5 µm
