# Supplementary material for: The dynamics of ciliogenesis in prepubertal mouse meiosis reveals new clues about testis maturation
Source: Front Cell Dev Biol. 2026 Aug 14;14:1910205. doi: 10.3389/fcell.2026.1910205 (PMC13522187; doi:10.3389/fcell.2026.1910205)
Supplement: Supplementary file 2 [file Image4.pdf]

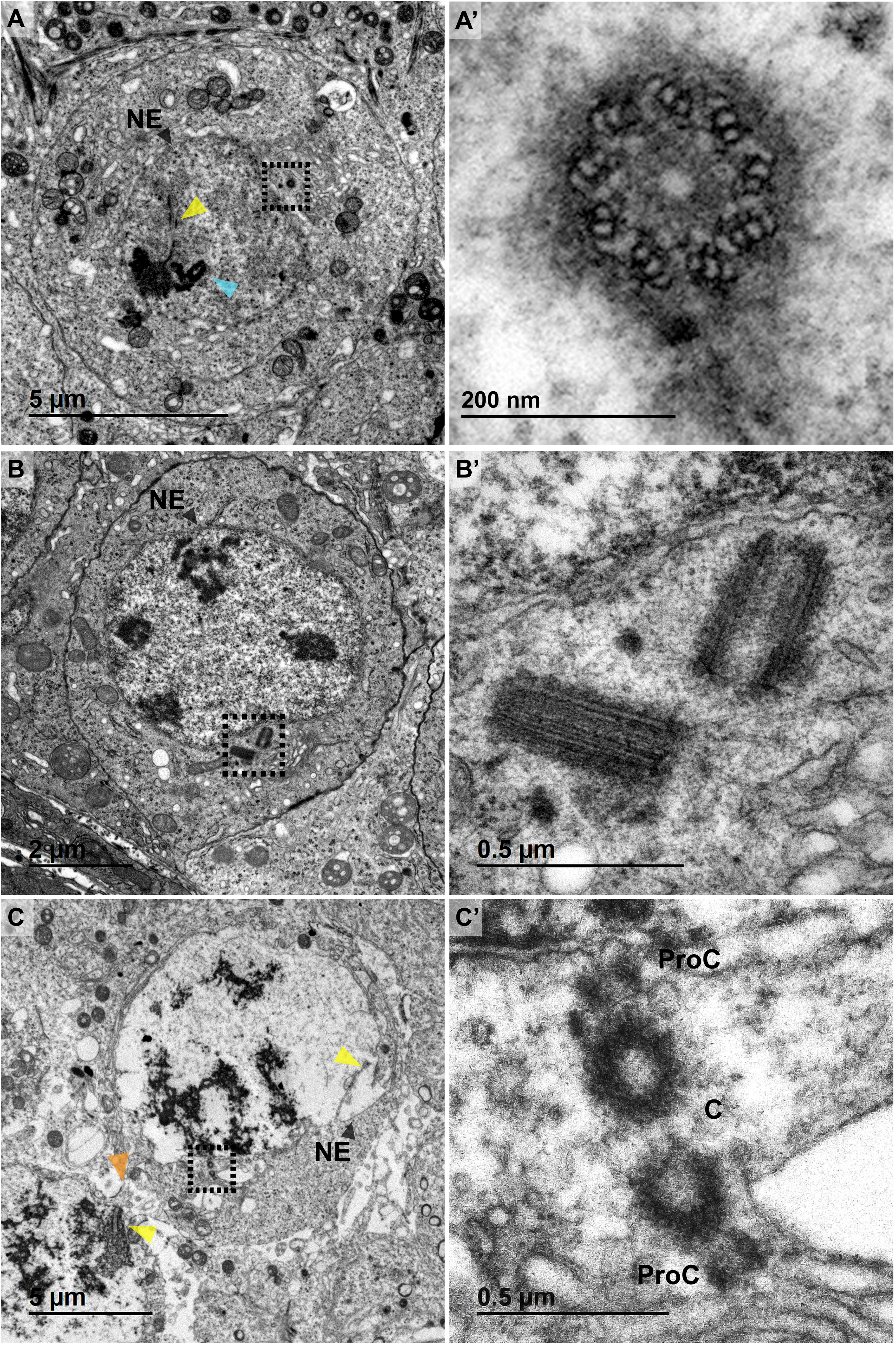

A
NE
5 µm
A'
200 nm
B
NE
2 µm
B'
0.5 µm
C
NE
5 µm
C'
ProC
C
ProC
0.5 µm
